# Vitamin D Receptor Mediates a Myriad of Biological Actions Dependent on Its 1,25‐Dihydroxyvitamin D Ligand: Distinct Regulatory Themes Revealed by Induction of Klotho and Fibroblast Growth Factor‐23

**DOI:** 10.1002/jbm4.10432

**Published:** 2020-12-03

**Authors:** Mark R Haussler, Sarah Livingston, Zhela L Sabir, Carol A Haussler, Peter W Jurutka

**Affiliations:** ^1^ Department of Basic Medical Sciences University of Arizona College of Medicine‐Phoenix Phoenix AZ; ^2^ School of Mathematical and Natural Sciences Arizona State University Glendale AZ

**Keywords:** PTH/VITAMIN D/FGF23, OSTEOBLASTS, OSTEOCYTES, TRANSCRIPTION FACTORS, BONE REMODELING/MOLECULAR PATHWAYS

## Abstract

The hormonal vitamin D metabolite, 1,25‐dihydroxyvitamin D [1,25(OH)_2_D], produced in kidney, acts in numerous end organs via the nuclear vitamin D receptor (VDR) to trigger molecular events that orchestrate bone mineral homeostasis. VDR is a ligand‐controlled transcription factor that obligatorily heterodimerizes with retinoid X receptor (RXR) to target vitamin D responsive elements (VDREs) in the vicinity of vitamin D‐regulated genes. Circulating 1,25(OH)_2_D concentrations are governed by PTH, an inducer of renal D‐hormone biosynthesis catalyzed by CYP27B1 that functions as the key player in a calcemic endocrine circuit, and by fibroblast growth factor‐23 (FGF23), a repressor of the CYP27B1 renal enzyme, creating a hypophosphatemic endocrine loop. 1,25(OH)_2_D/VDR–RXR acts in kidney to induce Klotho (a phosphaturic coreceptor for FGF23) to correct hyperphosphatemia, NPT2a/c to correct hypophosphatemia, and TRPV5 and CaBP28k to enhance calcium reabsorption. 1,25(OH)_2_D‐liganded VDR–RXR functions in osteoblasts/osteocytes by augmenting RANK‐ligand expression to paracrine signal osteoclastic bone resorption, while simultaneously inducing FGF23, SPP1, BGLP, LRP5, ANK1, ENPP1, and TNAP, and conversely repressing RUNX2 and PHEX expression, effecting localized control of mineralization to sculpt the skeleton. Herein, we document the history of 1,25(OH)_2_D/VDR and summarize recent advances in characterizing their physiology, biochemistry, and mechanism of action by highlighting two examples of 1,25(OH)_2_D/VDR molecular function. The first is VDR‐mediated primary induction of Klotho mRNA by 1,25(OH)_2_D in kidney via a mechanism initiated by the docking of liganded VDR–RXR on a VDRE at −35 kb in the mouse Klotho gene. In contrast, the secondary induction of *FGF23* by 1,25(OH)_2_D in bone is proposed to involve rapid nongenomic action of 1,25(OH)_2_D/VDR to acutely activate PI3K, in turn signaling the induction of MZF1, a transcription factor that, in cooperation with c‐ets1‐P, binds to an enhancer element centered at −263 bp in the promoter‐proximal region of the mouse *fgf23* gene. Chronically, 1,25(OH)_2_D‐induced osteopontin apparently potentiates MZF1. © 2020 The Authors. *JBMR Plus* published by Wiley Periodicals LLC on behalf of American Society for Bone and Mineral Research.

## The Vitamin D Receptor

### History

The vitamin D receptor (VDR) was discovered in 1967 in the laboratory of the late Anthony W. Norman, a pioneering researcher in the vitamin D field to whom this issue is dedicated. The first author (MRH) was a graduate student in Dr. Norman's laboratory at that time; we published our observation, “The Association of a Metabolite of Vitamin D_3_ With Intestinal Mucosa Chromatin, In Vivo” in 1968.^(^ ^1^ ^)^ This seminal finding was significant for two reasons: (i) after intracardially injecting radioactively labeled vitamin D into vitamin D‐deficient chickens, the predominant subcellular fraction of the target small intestine containing the radioactive tag was purified nuclear chromatin, revealing a role for DNA‐driven gene transcription in the molecular response to vitamin D; and (ii) when the chromatin‐associated, labeled sterol was extracted from intestinal chromatin and analyzed chromatographically in numerous systems, it proved to be a metabolite of vitamin D more polar than 25‐hydroxyvitamin D_3_ [25(OH)D_3_] and, like 25(OH)D_3_, biologically active.^(^ ^1^ ^)^ These insights illuminated that vitamin D was metabolized to a hormone‐like sterol, which comprises the functional form of vitamin D located at the actual target site in the proximity of DNA. In essence, this study from the Norman laboratory constituted the first appreciation/discovery of the functionally active metabolite of vitamin D, later chemically characterized as 1α,25‐dihydroxyvtamin D_3_.^(^ ^2^, ^3^ ^)^ Also in 1968, the more polar D metabolite first recognized in the Norman laboratory was discovered to be bound with high affinity and specificity to a protein that directed the novel D metabolite to intestinal chromatin and mediated its function in the genome. As reported in January 1969,^(^ ^4^ ^)^ a “chromosomal receptor for a vitamin D metabolite” had been brought to light, thus uncovering the missing link between this vitamin D metabolite and the genomic DNA of its biological target organ(s). This research is described in detail in the first author's (MRH) doctoral dissertation.^(^ ^5^ ^)^ For complex reasons,^(^ ^6^ ^)^ the initial work from the Norman laboratory on VDR was not widely embraced by the scientific community until Brumbaugh and colleagues^(^ ^7^, ^8^, ^9^, ^10^, ^11^, ^12^ ^)^ biochemically and pharmacologically defined the vitamin D receptor in an indisputable fashion.^(^ ^13^ ^)^ VDR became recognized as a member of the nuclear receptor superfamily, specifically the subfamily of ligand‐controlled transcription factors that obligatorily heterodimerize with retinoid X receptor (RXR) to bind cognate hormone responsive elements in DNA.^(^ ^14^ ^)^ In retrospect, the original observations in the Norman laboratory were truly the scientific breakthroughs that launched VDR as one of the 48 classic nuclear receptors encoded in the human genome and portended the emergence of this family of ligand‐activated transcription factors as functional regulators of gene expression that are essential in human biology and health, while also laying the groundwork for the central role of VDR in a myriad of biological actions that are still being discovered and appreciated today.

### Advances in VDR research

**Fig 1:**
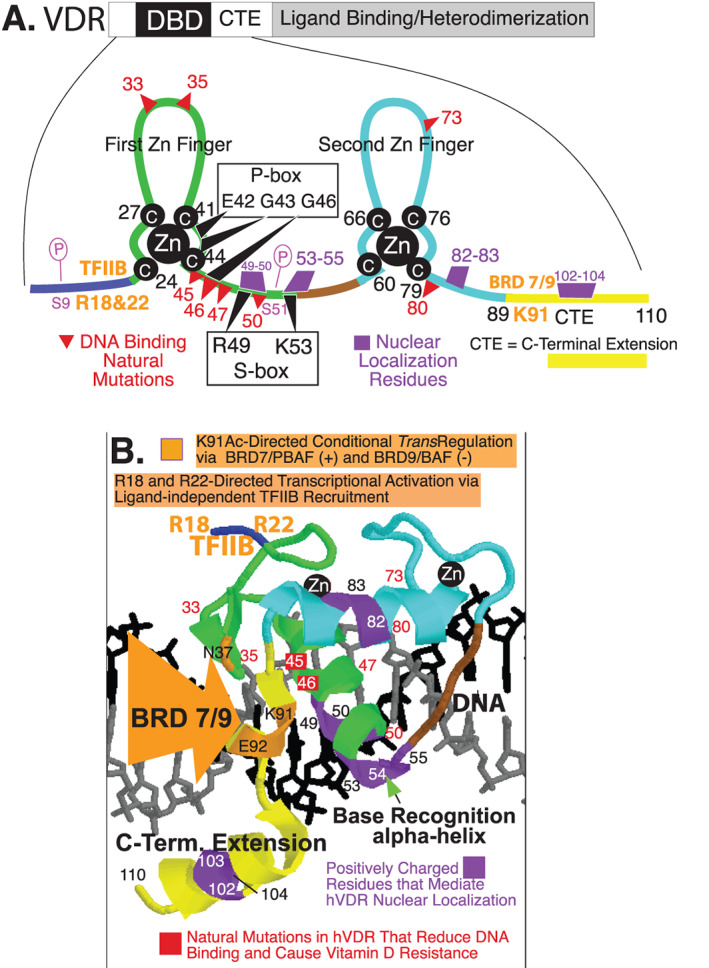
Human VDR DNA‐binding domain. (*A*) VDR schematic with the DBD/CTE magnified illustrating the two zinc fingers, DNA contact residues (P‐box and S‐box), DNA binding loss of function natural mutations, nuclear localization residues, transactivation residues (arginines 18 and 22; lysine 91), and phosphorylation sites (serines 9 and 51). (*B*) Model of VDR DBD/CTE bound to DNA. CTE = C‐terminal extension; DBD = DNA binding domain; VDR = vitamin D receptor; Zn = zinc.

Subsequent to its discovery and validation, there were numerous advances in VDR research that solidified the position of VDR and its 1α,25‐dihydroxyvtamin D_3_ ligand in the domains of molecular and clinical endocrinology. The initial quantum leap was achieved by J. Wesley Pike and colleagues who isolated avian VDR using DNA‐cellulose affinity chromatography and generated the first serum and monoclonal antibodies to the VDR protein.^(^ ^15^, ^16^ ^)^ This advance led to the molecular cloning of avian VDR by McDonnell and colleagues^(^ ^17^ ^)^ and to the cloning and expression of full‐length cDNA encoding the human vitamin D receptor.^(^ ^18^ ^)^ Thus, VDR joined the ranks of steroid and thyroid hormone/fat soluble vitamin nuclear receptors, structurally speaking, and it was the first member of this group to define the two zinc fingers as zinc atoms tetrahedrally coordinated by eight cysteine residues,^(^ ^19^ ^)^ establishing the DNA binding region of this (and all) nuclear receptors. Human VDR is depicted schematically at the top of Fig. 1*A*; the double zinc finger DNA binding domain (DBD) plus its C‐terminal extension (CTE) are pictured linearly in the balance of Fig. 1*A*. Shaffer and Gewirth crystallized a DNA‐binding fragment of human VDR ranging from residue 16 (phenylalanine) to 125 (serine) and determined its protein structure, reporting that it contained the following three α‐helices: (i) C‐41 to K‐53, (ii) Q‐77 to I‐87, and (iii) D‐97 to R‐121.^(^ ^20^ ^)^ These three structures are positioned on the right side of zinc fingers #1, #2, and in the distal CTE, respectively. They are not demarcated in Fig. 1*A*, but play essential roles in the binding of VDR to the 3′ half‐element of the VDRE in vitamin D‐regulated genes as modeled in Fig. 1*B*. The base‐recognition VDR α‐helix, namely C‐41 to K‐53, is pictured in green in Fig. 1*B*, nestled in the major groove of DNA where E42, K45, R49, and R50 contact the AGGTCA 3′ half‐element of a VDRE. This α‐helix contains both the proximal box (P‐box) and the specificity box (S‐box), groups of residues that direct the binding of VDR to estrogen response element‐type half‐elements (AGGTCA) over glucocorticoid response element‐type half‐elements (AGAACA), and possess an arginine (position 49 in human VDR) that specifies heterodimeric binding to direct repeat hormone‐responsive elements, respectively.^(^ ^21^ ^)^ A final DNA sequence recognition amino acid in this VDR α‐helix is lysine‐53, which, in conjunction with the spacing of basic residue clusters in the CTE, confers selective DR3 VDRE docking capacity on VDR.^(^ ^21^ ^)^ Therefore, as can be seen in Fig. 1*A*, the C‐41 to K‐53 proximal α‐helix in VDR constitutes the DNA sequence‐recognition command and control nexus of the receptor protein that directs 1,25(OH)_2_D/VDR to vitamin D targets in the genome.

**Fig 2:**
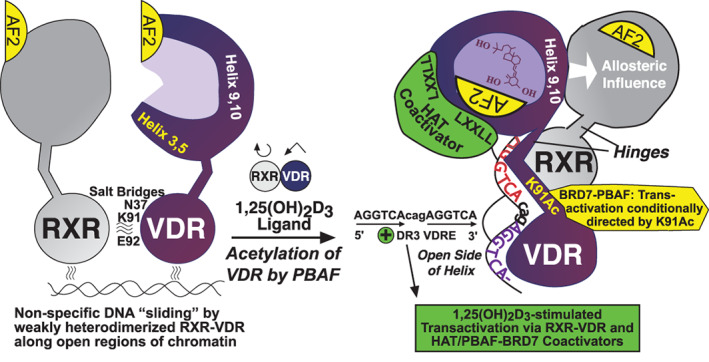
Molecular model for 1,25(OH)_2_D ligand‐dependent activation of VDR‐RXR, specific association of 1,25(OH)_2_D bound VDR‐RXR with a VDRE in DNA, followed by 1,25(OH)_2_D‐stimulated transactivation supported by novel comodulators. BAF = BRG1‐associated factors; CTE = C‐terminal extension; HAT = histone acetyl transferase; PBAF = polybromo‐associated BAF; RXR = retinoid X receptor; VDR = vitamin D receptor.

As illustrated in Fig. 1*B*, the second α‐helix in VDR (pictured in light blue) situated on the C‐terminal side of the second zinc finger is positioned to interact with the DNA phosphate backbone, and there is extensive contact between residues in this α‐helix (specifically R73, R74, and R80) and the phosphates of DNA. The combined energy provided by the DNA base recognition and phosphate backbone binding α‐helices is required for specific VDRE binding by VDR, and this conclusion is supported by the location of VDR point mutations (highlighted in red in Fig. 1) that are found in patients with hereditary hypocalcemic vitamin D‐resistant rickets.^(^ ^22^ ^)^ To bind to DNA and regulate gene expression, transcription factors depend upon nuclear translocation after biosynthesis on the polyribosomes, and nuclear localization mandates various arrangements of basic amino acids in short stretches within the primary amino acid sequence of the protein.^(^ ^23^ ^)^ Four such segments of positively charged amino acids that mediate unliganded VDR nuclear translocation exist in the DNA‐binding domain.^(^ ^24^, ^25^ ^)^ These residues align on one surface of VDR with its CTE accessible, extending along the structure of heterodimeric VDR–RXR according to calculations by Orlov and colleagues,^(^ ^26^ ^)^ creating an interaction surface for nuclear import factors initially, and subsequent to 1,25(OH)_2_D‐liganding and high‐affinity VDRE binding, contact with comodulators. The exception to this sequence of events is basic residues R‐49 and R‐50 in VDR, which first participate in nuclear localization, followed by VDRE base contact, and are therefore not involved in cofactor interaction. Prufer and colleagues^(^ ^27^ ^)^ have verified the essential role of arginine residues 49 and 50, as well as lysine‐53, arginine‐54, and lysine‐55 in VDR nuclear transfer, and shown that positionally equivalent basic residues in RXR are critical in nuclear localization, especially of the unliganded VDR‐RXR heterodimer.^(^ ^27^ ^)^ Thus, it appears that a quasi‐stable, hormone‐unoccupied VDR–RXR heterodimer is the species of receptor that localizes in the nucleus of vitamin D target cells, likely bound nonspecifically to DNA with relatively low affinity to slide along in open regions of chromatin (Fig. 2).

### A revelation on VDR control of transcription rooted in the DNA‐binding domain

To summarize, pictured in Fig. 1 are the following features: (i) first zinc finger in green, (ii) second zinc finger in light blue, (iii) cysteine residues that tetrahedrally coordinate the two zinc atoms— all in white type on black circles, (iv) some of the naturally occurring mutations that reduce DNA binding and cause the phenotype of vitamin D resistance in red, (v) nuclear localization residues in purple, (vi) C‐terminal extension in yellow, and (vii) N‐terminal region in dark blue. Finally, depicted in Fig. 1 are two transactivation motifs embedded in the VDR DBD that are colored orange. The N‐terminal transcriptional activation motif consists of arginine‐18 and arginine‐22, where the basal transcription factor TFIIB binds and augments mRNA synthesis from the target gene.^(^ ^28^ ^)^ Lysine‐91 emerged early on as a pivotal residue in VDR mechanism of action studies, particularly because its point mutation abolishes the transcriptional activation function of VDR and renders the mutant a dominant‐negative receptor.^(^ ^29^ ^)^ Very recently, a novel transcriptional activation motif centered on lysine‐91 was reported in the VDR CTE.^(^ ^30^ ^)^ In their study of 1,25(OH)_2_D/VDR and β cells of the endocrine pancreas, the research teams of Downes and Evans^(^ ^30^ ^)^ found that 1,25(OH)_2_D occupation of VDR switched comodulator complexes bound to K‐91 of VDR from bromodomain‐containing protein (BRD)9/BAF (BRG1‐associated factors), a repressive complex that conferred closed chromatin when VDR was unliganded, to BRD7/PBAF (polybromo‐associated BAF) in the presence of 1,25(OH)_2_D, which opened chromatin to transcriptional activation of a series of target genes whose gene products preserved β‐cell integrity. In the progression of type 2 diabetes mellitus, β‐cell damage and decay resulting from inflammatory stress triggered by insulin resistance that leads to endocrine pancreas exhaustion play a significant pathological role. In a quest to understand molecular factors that contribute to β‐cell exhaustion under diabetic conditions, Wei and colleagues^(^ ^30^ ^)^ carried out a genomic CRISPR (clusters of regularly interspaced short palindromic repeats) knockout screen of human‐induced pluripotent stem cell‐derived β‐like cells employing the methodology of Yoshihara and colleagues.^(^ ^31^ ^)^ By incorporating an inducible Cas9 expression system into β‐like cells bearing a human insulin promoter‐driven green fluorescent protein (GFP) reporter, Wei and colleagues^(^ ^30^ ^)^ were able to identify genes essential for β‐like cell maintenance. Pertinent to the present review, among the many genes revealed was that encoding human VDR, thereby illuminating the vitamin D hormone receptor as an essential regulator of inflammation and β‐cell survival. Further, alternative recognition of an acetylated lysine‐91 in human VDR by BRD7 and BRD9 appears to orchestrate association of PBAF and BAF chromatin remodeling complexes, respectively, with K‐91 in the CTE. Thus, K‐91 is a VDR residue that is posttranslationally acetylated,^(^ ^30^ ^)^ and this mechanistic development adds one more feature to the incredible potential of the VDR DBD: namely ligand‐dependent transcriptional control in stem cells, opening‐up a potential treasure trove of fundamental actions of VDR that are distinguished from the well‐established transcriptional activation mechanism of action involving the C‐terminal activation function‐2 (AF‐2) domain in VDR and recruitment of histone acetyl transferase (HAT) coactivators. The fact that 1,25(OH)_2_D ligand promotes VDR association with PBAF to effect genomewide changes in chromatin accessibility and enhancer landscape, resulting in an anti‐inflammatory response, is a novel feature of VDR–RXR function, but it may also be relevant to understanding the uncharacterized actions of the unliganded complex, such as in hair cycling. In other words, given that hair cycling in rodents and mammals is governed by unoccupied VDR,^(^ ^32^ ^)^ it is conceivable that ligandless VDR, bound to K‐91 in the VDR CTE, recruits repressive comodulators such as BRD9/BAF and Hairless^(^ ^33^ ^)^ to govern the mammalian hair cycle. One caveat to this proposal is that Wei and colleagues^(^ ^30^ ^)^ did not actually show experimentally that unoccupied VDR–RXR was bound to genes in their genomewide screen.

### VDR structure and a model for 1,25(OH)_2_D/VDR–RXR activation of transcription

The structure of 1,25(OH)_2_D‐occupied human VDR, heterodimerized with full‐length RXRα, docked on a VDRE and bound with a single coactivator, has been determined in solution via small‐angle X‐ray scattering and fluorescence resonance energy‐transfer techniques,^(^ ^34^ ^)^ as well as by cryoelectron microscopy (cryo‐EM).^(^ ^26^ ^)^ These two advances, plus additional structural investigation of the VDR–RXR heterodimer,^(^ ^35^ ^)^ permit the creation of a visual model showing how the DBD and the ligand binding/heterodimerization domains of VDR and RXR are arranged relative to one another, and how their binding to cognate ligand, DNA, and coactivators may influence one another. The left side of Fig. 2 is a hypothetical depiction of unoccupied VDR and RXR, weakly heterodimerized via salt bridges from the RXR DBD to human VDR residues N‐37, K‐91, and E‐92, having already been translocated to the nucleus as discussed above. The quasi‐stable complex shown on the left is postulated to be transiently bound and “sliding” nonspecifically along DNA in open regions of chromatin. The VDR is depicted as unoccupied on the left side of Fig. 2, with the AF‐2 α‐helix 12 in the open configuration awaiting 1,25(OH)_2_D ligand penetration of the hydrophobic pocket of the VDR‐ligand–binding domain. The right side of Fig. 2 illustrates in schematic fashion the spatial organization for a 1,25(OH)_2_D‐liganded VDR–RXR heterodimer bound to a generic VDRE DR3 element. This representation is adapted from Orlov and colleagues,^(^ ^26^ ^)^ and shows the RXR hetero‐partner unoccupied by its 9‐cis retinoic acid ligand despite the fact that Orlov and colleagues^(26)^ included an excess of the retinoid along with 1,25(OH)_2_D to stabilize the protein structures for physical characterization. Our model excludes the RXR ligand based on biochemical data, indicating that 9‐cis retinoic acid suppresses rather than enhances 1,25(OH)_2_D‐triggered transactivation by VDR,^(^ ^36^ ^)^ and we have accumulated evidence that 1,25(OH)_2_D association with VDR not only amplifies dimerization with RXR, but also allosterically influences the AF‐2 helix 12 of RXR to assume the closed conformation for coactivator recruitment.^(^ ^37^, ^38^ ^)^ Therefore, the key event in VDR‐mediated gene activation is the binding of the 1,25(OH)_2_D ligand, which generates a dramatic conformational change in the position of helix 12 at the C‐terminus of VDR, bringing it to the “closed” position to serve as part of a platform for binding the LxxLL domains of coactivators.^(^ ^39^, ^40^ ^)^ The binding of coactivator, in turn, likely stabilizes the VDR–RXR heterodimer on the VDRE, and may even assist in heterodimerization by conformationally inducing the VDR LBD to face the open side of the DNA helix at the 5′ end of the VDRE (see right portion of Fig. 2). The AGGTCA*cag*AGGTCA DR3 VDRE is also shown in Fig. 2 as is the K‐91–containing CTE, which is positioned as contacting the *cag* spacer in the DR3 VDRE. The model of Orlov and colleagues^(26)^ also portrays the CTE of the VDR DBD as facing the open side of the DNA helix opposite the side occupied by the RXR DBD, indicating that the CTE engages in coactivator recruitment.^(^ ^41^ ^)^ Finally, in addition to the HAT coactivator bound to closed helix 12 on the helix 3 and 5 platform of liganded VDR as illustrated in Fig. 2, shown is the BRD7/PBAF‐coactivator complex that associates with lysine‐91 in the VDR CTE.^(^ ^30^ ^)^ These structural alterations and protein–protein associations upon 1,25(OH)_2_D‐binding are made possible by the flexibility imparted on VDR via its hinge domain,^(^ ^26^ ^)^ a segment of human VDR beginning at proline‐122, which appears to be endowed with other properties beyond VDR conformational flexibility such as: (i) binding to the nuclear matrix to effect a reduction of VDR‐mediated transcription in mitotic cells,^(^ ^42^ ^)^ and (ii) phosphorylateable serine/threonine residues in which the phosphorylation status affects the transcriptional activation capacity of the VDR. A specific example of the latter is human VDR hinge residue serine‐208,^(^ ^43^ ^)^ which is phosphorylated by casein kinase II in a posttranslational modification that potentiates receptor transcriptional activity,^(^ ^44^ ^)^ probably via reduced proteolytic degradation of VDR that promotes a suprafunctional concentration of receptor.

In conclusion, the DNA‐binding region of VDR is truly a domain that commands a myriad of biological functions, not only directing 1,25(OH)_2_D‐liganded VDR to the many specific VDREs in the genome while engaged in DNA surveillance, but possessing amino acid sequences that impart nuclear localization plus conditional transcriptional activation, as well as likely holding the key to the actions of unliganded VDR. The DBD is clearly the “business end” of the VDR molecule that is launched by the important functions of the ligand binding/heterodimerization domain, and its properties led to the discovery of VDR in 1968/1969^(^ ^1^, ^4^ ^)^ and to its purification in 1979^(^ ^45^ ^)^ and cloning in 1987.^(^ ^17^ ^)^

## VDR Mediates Vitamin D Control of Bone Mineral Physiology

### The calcium homeostasis endocrine loop

**Fig 3:**
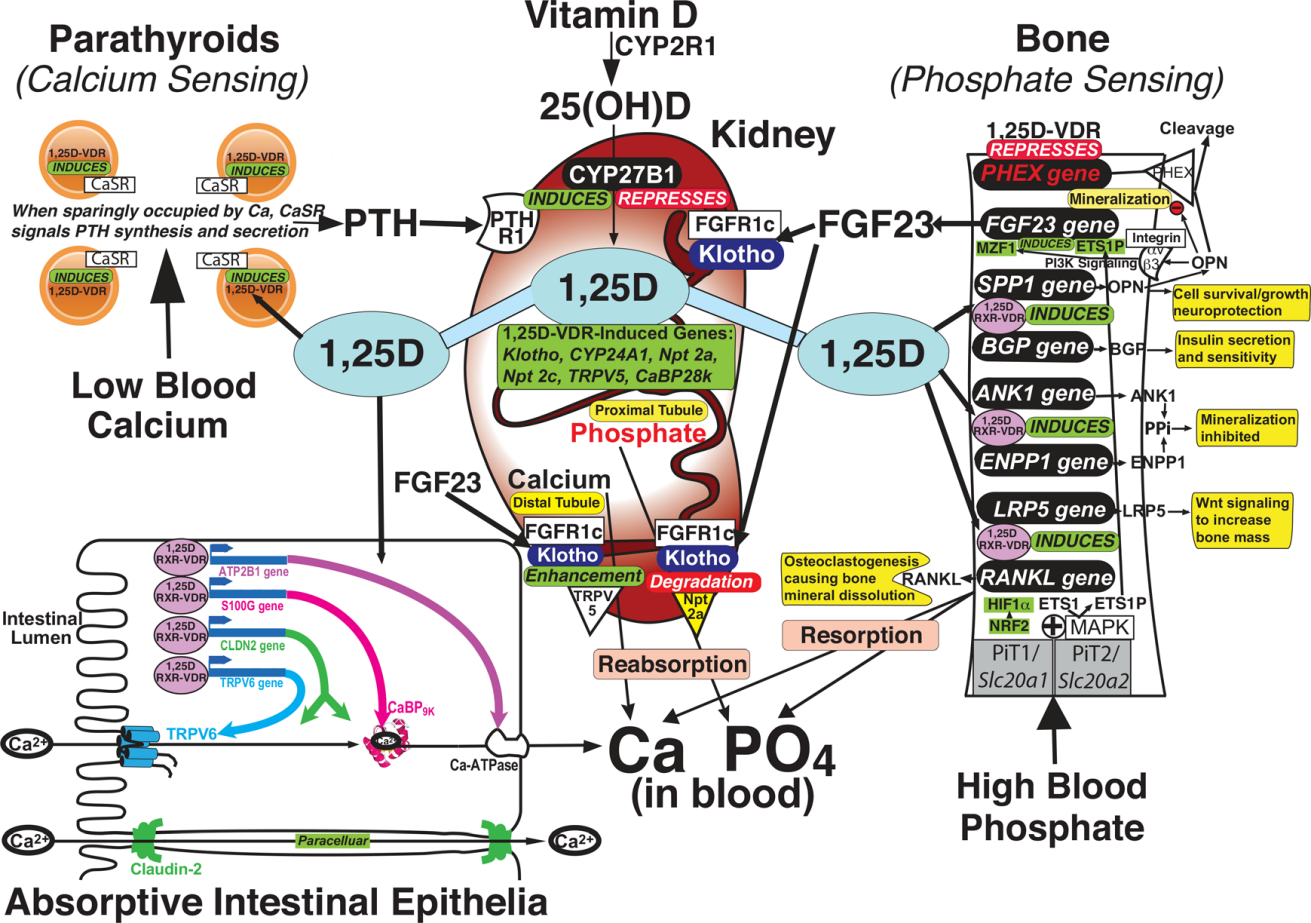
Vitamin D receptor mediates 1,25(OH)_2_D control of bone mineral physiology.

As illustrated in Fig. 3, the hormonal metabolite of vitamin D, 1,25(OH)_2_D, is produced predominantly in kidney, and acts in a variety of end organs via nuclear VDR to trigger an ensemble of molecular events that orchestrate bone mineral homeostasis. Circulating 1,25(OH)_2_D concentrations are governed by PTH,^(^ ^46^ ^)^ an inducer of renal D‐hormone biosynthesis catalyzed by CYP27B1 that functions as the key player in a calcemic endocrine loop, and fibroblast growth factor‐23 (FGF23), a repressor of the CYP27B1 renal enzyme that completes a novel hypophosphatemic endocrine loop.^(^ ^47^ ^)^ The calcium homeostatic loop, highlighted in Fig. 3, is triggered by low blood calcium, which signals via suboptimal‐occupation of the calcium‐sensing receptor (CaSR) to elicit the elaboration of PTH by the parathyroid glands. PTH then induces renal CYP27B1 to enhance the synthesis of 1,25(OH)_2_D, which acts in an autocrine manner in kidney to enhance calcium reabsorption from the glomerular filtrate via induction of TRPV5 and CaBP_28K_, and in endocrine fashion in the following primary vitamin D calcemic targets: intestine, bone, and parathyroid (Fig. 3). The resulting calcemia corrects calcium imbalance and closes the feedback loop through full occupation of the CaSR and silencing of PTH synthesis and secretion. In the process of correcting hypocalcemia, additional phosphate is mobilized by 1,25(OH)_2_D through absorption from the gut and resorption from bone, but this excess phosphate is eliminated acutely via PTH‐signaled phosphaturia and chronically through FGF23 action to inhibit renal phosphate reabsorption. Thus, bone mineral balance is maintained.

### The phosphate homeostasis endocrine loop

FGF23 arises mainly from osteocytes,^(^ ^48^ ^)^ rendering bone a newly recognized endocrine organ that is counter‐regulatory to PTH with respect to vitamin D and calcium metabolism, although both PTH and FGF23 are hypophosphatemic in that they signal the inhibition of renal phosphate reabsorption. The essence of the phosphate homeostatic loop is phosphate sensing by bone cells that governs FGF23 synthesis and secretion. As highlighted in Fig. 3, high blood phosphate signals via a putative phosphate sensor, the PiT1/*Slc20a1* and PiT2/*Slc20a2* heterodimer, to initiate MAPK stimulation that catalyzes a phosphorylation cascade producing activated transcription factor intermediaries in FGF23 induction. These transcription factors remain unidentified, although we propose that NRF2 and/or HIF1α are reasonable candidates based on evidence discussed in detail in this review. Once FGF23 is elaborated by the skeleton, it functions by binding to renal FGFR1c/Klotho coreceptors to promote phosphaturia,^(^ ^49^ ^)^ calcium reclamation,^(^ ^50^ ^)^ and repress CYP27B1^(^ ^51^ ^)^ as well as induce CYP24A1^(^ ^49^, ^52^ ^)^ (Fig. 3), with the latter two actions serving to curb 1,25(OH)_2_D levels. The resulting phosphaturia and circulating 1,25(OH)_2_D reduction correct the phosphate excess (while preserving serum calcium as a result of renal reabsorption) and close the feedback loop through diminished occupation of the postulated PiT1/*Slc20a1*‐PiT2/*Slc20a2* phosphate sensor, silencing FGF23 synthesis and secretion by osteocytes.

### Bone mineral endocrine physiology

Thus, through the mechanisms described above and detailed in Fig. 3, extracellular calcium and phosphate are maintained at physiologically normal levels by 1,25(OH)_2_D, PTH, and FGF23, facilitating appropriate bone mineralization and remodeling. 1,25(OH)_2_D, the vitamin D hormone, is the linchpin of an intricate endocrine network that sustains calcium and phosphate homeostasis to prevent bone disease. Notably, the reciprocity of calcium and phosphate metabolism reflects the physicochemical relationship of these two bone mineral ions, with the foundation of chronic regulation being the reciprocal control of CYP27B1 and CYP24A1 by PTH and FGF23. New mechanistic insight published by the Pike group with respect to endocrine control of 25D‐OHases, CYP27B1,^(47^ ^)^ and CYP24A1,^(^ ^53^ ^)^ therefore provides a solution to the molecular puzzle of how bone mineral ions are regulated in normal physiology. In a classic converse endocrine fashion, PTH from the parathyroid glands suppresses renal CYP24A1,^(^ ^47^ ^)^ the enzyme catalyzing the first step in 1,25(OH)_2_D elimination, and FGF23 from bone induces renal CYP24A1.^(^ ^53^ ^)^

### 1,25(OH)_2_D/VDR–RXR actions in the parathyroid gland, small intestine, and bone

1,25(OH)_2_D acts primarily at the small intestinal enterocyte to induce calcium absorption, but it also functions in parathyroid glands to induce the CaSR and amplify their calcium perceiving sensitivity, in osteoblasts/osteocytes in cooperation with phosphate to induce FGF23, and in kidney proximal tubules to auto‐regulate 1,25(OH)_2_D by inducing CYP24A1 and repressing CYP27B1. As depicted in Fig. 3, in small intestine, 1,25(OH)_2_D induces a number of gene products that mediate calcium absorption, including TRPV6, CaBP_9k_ (S100G), PMCA1b (ATP2B1), and CLDN2. 1,25(OH)_2_D also appears to increase expression of NPT2b in the small intestine to promote phosphate absorption, but this action on phosphate is not pictured in Fig. 3 because it may only be relevant under extreme conditions of phosphate restriction.^(^ ^54^ ^)^

As detailed in Fig. 3, 1,25(OH)_2_D/VDR acts directly in bone cells to impact skeletal metabolism in numerous respects, not only stimulating resorption to dramatically influence remodeling, but to modulate mineralization in far‐reaching mechanisms beyond simply supplying calcium and phosphate for mineral accretion. Accordingly, 1,25(OH)_2_D/VDR transcriptionally regulates a minimum of nine genes in bone, and these actions are sufficient to generate normal bone mineral physiology as currently understood. (i) 1,25(OH)_2_D/VDR induces RANKL^(^ ^55^ ^)^ (encoded by the *TNFSF11* gene) that promotes osteoclastogenesis from osteoclast precursor cells, culminating in bone mineral resorption. (ii) 1,25(OH)_2_D/VDR induces *LRP5*,^(^ ^56^ ^)^ the gene product of which stimulates Wnt signaling to increase bone mass. (iii, iv) 1,25(OH)_2_D/VDR induces *ANK1* and *ENPP1*,^(^ ^57^ ^)^ with both of their gene products enhancing pyrophosphate (PPi) concentrations that inhibit bone mineralization. (v) 1,25(OH)_2_D/VDR induces *BGLAP*,^(^ ^56^ ^)^ of which the gene product BGP/osteocalcin constitutes yet a third bone hormone (beyond FGF23 and osteopontin [OPN]) that functions systemically to increase insulin secretion by the β cells of the pancreas, insulin sensitivity of adipose and muscle, and to amplify spermatogenesis via the Leydig cells of the testes, as well as act to attenuate the parasympathetic nervous system to potentiate the “fight‐or‐flight” response. (vi) As reported in Fig. S1 *C*, 1,25(OH)_2_D/VDR induces *SPP1*, of which the gene product OPN functions systemically to promote cell survival and growth, as well as locally to curtail bone mineralization. This latter action of OPN is critical to the prevention of ectopic calcification, whereas the former function may elicit neuroprotection.^(^ ^58^, ^59^ ^)^ Indeed, VDR agonists induce both OPN and Klotho, while decreasing aortic calcification in mice with chronic kidney disease fed a high phosphate diet.^(^ ^60^ ^)^ (vii) 1,25(OH)_2_D/VDR represses *PHEX*,^(^ ^61^ ^)^ which encodes the exopeptidase that possesses a loss‐of‐function mutation in X‐linked hypophosphatemic rickets and operates enzymatically to cleave and eliminate the ability of OPN to retard bone mineral accretion, as well as to putatively function as the primarily induced protein that drives the extended secondary induction of FGF23 by 1,25(OH)_2_D (as posited below in the subsection, Osteopontin Controls Bone Mineral Deposition in Conjunction With FGF23). (viii) 1,25(OH)_2_D/VDR secondarily induces FGF23^(^ ^62^ ^)^ through a proposed OPN/αvβ3integrin/PI3K/MZF1 pathway, which appears to require c‐ETS1‐P as a cooperating transcription factor (see subsection, Osteopontin Controls Bone Mineral Deposition in Conjunction With FGF23). (ix) 1,25(OH)_2_D/VDR represses *RUNX2* ^(^ ^56^ ^)^ (not shown in Fig. 3), the master regulator of osteoblast differentiation and a controller of the expression of many bone genes.^(^ ^63^ ^)^ The bottom line is that by governing the expression of numerous genes, 1,25(OH)_2_D/VDR signals the sculpting and remodeling of bone mineral in situ much the way Michelangelo created the David. Finally, the above summarized pathways for endocrine control of the skeleton, and defects therein, essentially account for all molecular pathologies of bone mineral diseases that have been recognized to date. Because two major challenges faced by terrestrial animals are limited calcium availability that could compromise the endoskeleton and curtail mobility, along with an overabundance of phosphate in the diet that promotes aging and chronic disease, it is easy to comprehend the significance of the compensatory and corrective pathways executed by the vitamin D endocrine system as depicted in Fig. 3. Because VDR underpins the plethora of 1,25(OH)_2_D actions, the importance of this macromolecule to health and wellbeing cannot be underestimated. When liganded with 1,25(OH)_2_D, VDR–RXR directs a biochemical program that optimizes bone mineral usage to maintain the quality of life itself. The balance of this review of VDR will highlight the following two specific examples of its execution of 1,25(OH)_2_D molecular function: (i) primary induction of Klotho in kidney, and (ii) secondary induction of FGF23 in bone.

## Renal Klotho Regulation by 1,25(OH)_2_D Occurs via a Primary Genomic Mechanism

### The kidney is both the source of endocrine 1,25(OH)_2_D and a target for its actions

As we have emphasized in reviews over the past 20 years,^(^ ^32^, ^62^, ^64^, ^65^, ^66^, ^67^, ^68^, ^69^ ^)^ the kidney is the nexus of the vitamin D endocrine system: both generating 1,25(OH)_2_D in response to hypocalcemic signals and engaging as a site for metered bone mineral elimination and/or reabsorption. Fig. 3 illustrates the central role of the kidneys in the metabolic activation of vitamin D catalyzed by CYP27B1 in response to PTH‐elicited stimulation of the renal enzyme, normally under conditions such as hypocalcemia. The effects of endocrine 1,25(OH)_2_D are sufficient to generate normal bone mineral physiology as we now understand it, as well as to promulgate the plethora of other biological actions now attributed to 1,25(OH)_2_D/VDR–RXR. As also depicted in Fig. 3, 1,25(OH)_2_D/VDR‐RXR drives transcriptional activation of a minimum of six genes in kidney, including Klotho (*KL*), *NPT2a*, *NPT2c*, *TRPV5*, *CaBP*_*28K*_, and *CYP24A1*. Developed below is one focus of the current review, namely the relatively recent discovery that 1,25(OH)_2_D/VDR–RXR induces *KL* mRNA in the kidney.^(^ ^70^ ^)^ We also highlight the progress made in the last decade defining the pathobiological significance of Klotho and characterize the mechanism of its regulation by the vitamin D hormone.

### Renal Klotho expression is governed by 1,25(OH)_2_D/VDR–RXR binding to VDREs

**Fig 4:**
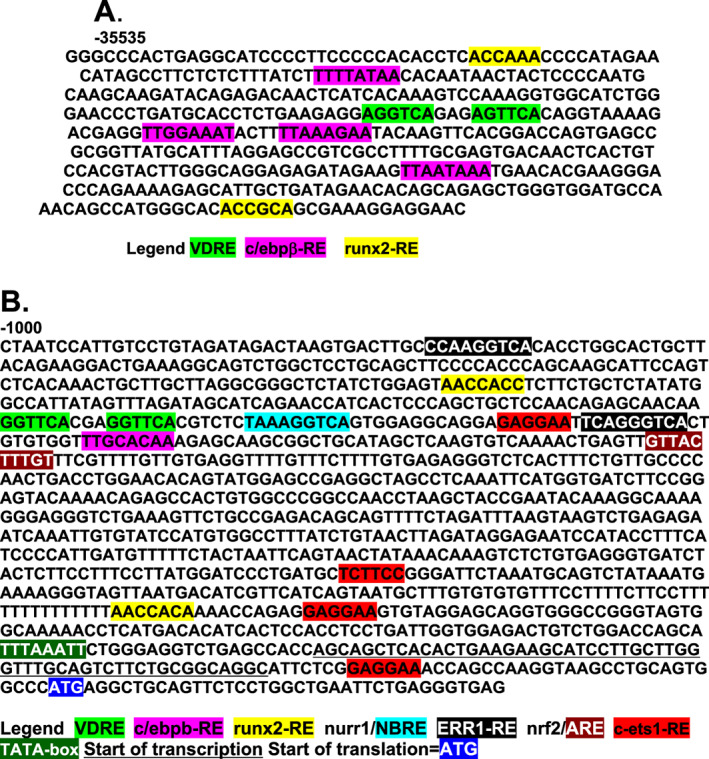
VDREs in two 1,25(OH)_2_D/VDR–RXR induced genes. (*A*) −35 kb region of the mouse *klotho* gene. (*B*) Promoter proximal region of the mouse *spp1*/osteopontin gene. VDREs = vitamin D responsive elements.

α‐Klotho (referred to herein as Klotho) was first reported by Kuro‐o and colleagues.^(^ ^71^ ^)^ Its disruption in mice is associated with soft tissue calcification, profound hyperphosphatemia, osteoporosis, emphysema, arteriosclerosis, skin atrophy, infertility, hypoglycemia, and a curtailed lifespan. A recessive inactivating mutation in the human *KL* gene elicits the phenotype of severe tumoral calcinosis.^(^ ^72^ ^)^ *KL* is expressed primarily in the kidneys and brain choroid plexus.^(^ ^73^ ^)^ With respect to the regulation of Klotho biosynthesis, Forster and colleagues^(^ ^70^ ^)^ reported that 1,25(OH)_2_D significantly induces mRNA expression of *KL* in a human renal (HK‐2) cell line and *kl* in a mouse distal convoluted tubule (mpkDCT) cell line. These findings indicate that 1,25(OH)_2_D is capable of both amplifying FGF23 responsiveness in the kidney by inducing the Klotho membrane coreceptor for FGF23 and of eliciting elaboration of the shed soluble Klotho hormone. To mechanistically probe regulation of *KL* by 1,25(OH)_2_D, Forster and colleagues^(^ ^70^ ^)^ performed bioinformatic analyses of both the human and mouse Klotho genes, which unveiled numerous candidate VDREs in mouse and human genes.^(^ ^70^ ^)^ When assessed for functionality by cotransfection of reporter constructs into HK‐2 cells, only one mouse VDRE (AGGTCAgagAGTTCA) located at −35,360 bp and two human VDREs (TGAACTctaCGAACC and TGAACTtctTGAACT) located on the negative strand at −47,293 bp and −32,203 bp, respectively, displayed a potency similar to the established rat osteocalcin VDRE.^(^ ^70^ ^)^ Notably, the –35 kb area of the mouse Klotho gene given in Fig. 4*A* is marked by VDR and RXR in vivo as deciphered by ChIP‐seq technology (J. Wesley Pike, personal communication, November 24, 2017). Furthermore, a ChIP‐seq map of the human genome^(^ ^74^ ^)^ yields an additional pair of VDREs, AGTTGAaagGGTTCC and GGAACTgcaTCCACC (negative strand), in the first intron of *KL* at +1536 and + 1392 bp, respectively. We thus propose that 1,25(OH)_2_D‐liganded VDR–RXR induces Klotho expression by binding to functional VDREs somewhat remote to the mouse and human *KL* structural genes, with the human VDREs apparently being separated by as much as 49 kb. Importantly, the VDREs identified in *KL* genes fit the pattern of possessing nearby cis‐elements that bind c/ebpβ and runx2 (Fig. 4*A* is an illustration of this for the mouse Klotho VDRE), a signature property of many 1,25(OH)_2_D/VDR‐RXR‐induced genes,^(^ ^75^ ^)^ most recently highlighted by the case of *mmp13*.^(^ ^76^ ^)^ Finally, as presented in Fig. 4*B* for comparison of VDRE environments, the promoter proximal region of the mouse *spp1* gene reveals a classic VDRE that is flanked on the 5′‐side by a runx2 site and on the 3′‐side by a c/ebpβ element. In combination with the data of Tsujikawa and colleagues,^(^ ^77^ ^)^ that 1,25(OH)_2_D increases steady‐state Klotho mRNA levels in mouse kidney in vivo, the results reviewed herein verify that 1,25(OH)_2_D is the first discovered natural inducer of the *Klotho* longevity gene. Moreover, Tsujikawa and colleagues^(^ ^77^ ^)^ showed that renal *Klotho* mRNA induction after 1,25(OH)_2_D injection displayed identical kinetics to those of renal *cyp24a1*, a well‐established primary induction target for 1,25(OH)_2_D/VDR. In summary, Klotho is controlled by 1,25(OH)_2_D via a primary VDR–RXR mechanism consisting of direct binding of the hormone‐receptor complex to bona fide VDREs remote from the structural gene, but each positioned in the immediate neighborhood of cis‐associated c/ebpβ and runx2 cooperating transcription factors that apparently generate multiprotein complexes to drive induction of *KL* mRNA biosynthesis.

### Klotho and its actions on ion transport in the kidney

The gene products for *KL* consist of multiple Klotho protein forms including a full‐length transmembrane Klotho, a proteolytically shed soluble Klotho, and a much less‐abundant secreted truncated Klotho with unknown function that may not exist in humans because of degradation of alternatively spliced *Klotho* mRNA.^(^ ^78^ ^)^ The transmembrane Klotho (mKL) form is cleaved by membrane‐anchored proteases including ADAM17, liberating the shed soluble Klotho (sKL) that contains both KL1 and KL2 domains functional in FGF23 ligand binding by the coreceptor complex of sKL and FGFR1c. After entering the circulation, this shed soluble Klotho form may function as a circulating hormone‐like principle.^(^ ^78^ ^)^ This circulating Klotho form has been reported to employ the glycosyl hydrolase catalytic activity to regulate the TRPV5 calcium channel^(^ ^79^ ^)^ and the renal outer medullary potassium channel 1 (ROMK1).^(^ ^80^ ^)^ However, in a recent review, Erben^(^ ^50^ ^)^ summarized the work of Chen and colleagues,^(^ ^81^ ^)^ who crystallized the ternary complex of the extracellular domain of FGFR1c, FGF23 ligand, and the Klotho ectodomain to provide compelling experimental evidence that Klotho lacks any biologically relevant glycosidase activity. Thus, there has occurred a recent paradigm shift in our understanding of Klotho to reveal that it is devoid of enzymatic activity and instead increases the abundance of TRPV5 on the renal cell membrane through FGF23 signaling (Fig. 3) via ERK1/2, SGK1, and WNK4 to depress endocytotic removal of TRPV5 from the plasma membrane.^(^ ^50^ ^)^ Thus, 1,25(OH)_2_D, by independently inducing TRPV5^(^ ^82^ ^)^ and Klotho,^(^ ^70^ ^)^ apparently elicits FGF23‐driven calcium retention at the kidneys. One could speculate, therefore, that 1,25(OH)_2_D/VDR–RXR chronically initiates calcium conservation at the kidneys over a lifetime, possibly to lower the incidence of osteoporosis. ROMK1 is likely upregulated by Klotho analogously to TRPV5 through enhancement in the apical membrane via ERK1/2, SGK1, and WNK4‐signaling, but the outcome would be increased potassium excretion.

### Klotho is a FGF23 coreceptor in controlling phosphate and vitamin D metabolism

The most physiologically significant function of full‐length, membrane Klotho is to act as a renal coreceptor of FGFR1c in the feedback control of phosphate and vitamin D metabolism by bone‐derived FGF23 (Fig. 3). FGF23 signals by binding to renal FGFR1c/Klotho coreceptors and an ERK/SGK1/NHERF1 phosphorylation‐transduction pathway to cause degradation of membrane sodium phosphate cotransporter type 2a (Npt2a), with resultant downregulation of phosphate reabsorption to promote phosphaturia.^(^ ^49^ ^)^ FGF23 also signals to repress renal specific CYP27B1^(^ ^51^ ^)^ via a pathway that involves the binding of as yet unidentified transcription factors (triggered by ERK1/2) to upstream regions of the mouse *cyp27b1* gene centered at approximately −5 kb and −12.5 kb.^(^ ^47^ ^)^ Finally, FGF23 further signals to induce renal CYP24A1 by binding to FGFR1c/Klotho coreceptors and promulgating an as yet unknown signal transduction pathway initiated by ERK1/2 and including unidentified transcription factors that bind to a downstream region of the mouse *cyp24a1* gene centered at approximately +27 kb and possibly to the promoter proximal sequence immediately upstream of the transcription start site,^(^ ^53^ ^)^ resulting in induction of CYP24A1.^(^ ^49^, ^52^ ^)^ The latter two actions serve to curb 1,25(OH)_2_D levels (Fig. 3). Remarkably, double knockouts of FGF23 (or its Klotho coreceptor) with either VDR^(^ ^83^ ^)^ or CYP27B1^(^ ^84^ ^)^ essentially rescue FGF23 null mice, underscoring the role of FGF23 and Klotho as counter‐regulatory hormones to 1,25(OH)_2_D, which appears to be the key to their health and longevity benefits. Shed, soluble Klotho may possess systemic antiaging properties independent of the phosphaturic and 1,25(OH)_2_D‐attenuating actions of transmembrane Klotho, but the mechanism(s) of these actions is not known.^(^ ^80^ ^)^ Conversely, although FGF23 is antiaging at the kidney by eliciting phosphate elimination and detoxifying 1,25(OH)_2_D, its “off‐target” actions could actually be pro‐aging in terms of coronary artery disease, as well as potential neoplastic actions in the colon,^(^ ^85^ ^)^ and it is possible that these off‐target FGF23 pathologies are “buffered” by soluble Klotho.^(^ ^86^ ^)^ Upregulation of Klotho by 1,25(OH)_2_D^(^ ^70^ ^)^ is thus consistent not only with potentiation of FGF23 signaling in the kidney, but also may offer protection for other cell types (eg, vascular and colon), in which the circulating soluble form of Klotho could exert beneficial actions.^(^ ^87^ ^)^

### Klotho exerts numerous additional bioprotective and cellular antiaging actions

Klotho exerts antioxidative effects, some of which are reminiscent of 1,25(OH)_2_D actions. For example, Klotho has been reported to bind to the transient‐receptor potential canonical Ca^2+^ channel 1 (TRPC‐1) through its KL2 domain, and regulates TRPC‐1‐mediated Ca^2+^ entry to maintain endothelial integrity and prevent Ca^2+^‐stimulated nitric oxide synthetase formation, which contributes to the formation of potent reactive nitrogen species.^(^ ^88^ ^)^ Thus, the maintenance of Ca^2+^ and redox signaling at a low resting state by 1,25(OH)_2_D and its effectors, Nrf2 and Klotho, appears to constitute the mechanism whereby 1,25(OH)_2_D and Klotho prevent the ravages of oxidation. Also, Wang and colleagues^(^ ^89^ ^)^ reported that Klotho downregulates the expression of a catalytic subunit of NADPH oxidase and suppresses angiotensin II‐induced superoxide production, oxidative damage, and apoptosis through the cAMP/PKA pathway. In vivo, Klotho gene delivery similarly attenuates NADPH oxidase activity and superoxide production to prevent the progression of spontaneous hypertension and resulting renal damage.^(^ ^87^ ^)^ These findings led us to propose that 1,25(OH)_2_D/VDR–RXR primary induction of Klotho mRNA represents a natural pathway to maintaining healthful aging, with intracellular calcium current regulation and mitigation of oxidation being common themes.

Klotho is also known to impact Wnt signaling^(^ ^73^ ^)^ to curtail renal fibrosis and perhaps suppress tumorigenesis. Klotho‐mediated regulation of Wnt signaling was reported by Liu and colleagues,^(^ ^90^ ^)^ who showed that sKL binds to Wnt ligands to suppress downstream signal transduction, and that *KL* knockout enhances Wnt signaling in mice. Regarding the disease‐related consequences of Wnt signaling suppression by Klotho, activated Wnt3 signaling extends the cell cycle by arresting it at the G2/M phase, and induces fibrogenic cytokines in mouse kidney; but Klotho‐treated cells circumvent this phase and are protected against renal fibrosis.^(^ ^91^ ^)^ The loss of Klotho may therefore contribute to kidney injury by releasing the inhibition of pathogenic Wnt/β‐catenin signaling.^(^ ^92^ ^)^ Indeed, in vivo expression of Klotho decreases the activation of renal β‐catenin and diminishes renal fibrosis in chronic kidney disease.^(^ ^92^ ^)^ Conversely, reduced Klotho expression aggravates renal interstitial fibrosis,^(^ ^93^ ^)^ and overexpression of sKL abolishes the fibrogenic effects of TGF‐β1.^(^ ^92^, ^94^ ^)^ In summary, Klotho overexpression or supplementation protects against fibrosis in several models of renal and cardiac fibrotic disease, with its actions appearing to be rooted in the direct inhibitory effects of circulating/soluble Klotho on TGFβ1 and Wnt signaling.^(^ ^95^ ^)^

Regarding antitumor actions of Klotho, Behera and colleagues^(^ ^96^ ^)^ found a correlation between loss of Klotho and a gain in Wnt5A expression, leading to progression of melanoma. Similarly, Abramovitz and colleagues^(^ ^97^ ^)^ have reported that both membrane and soluble Klotho serve as tumor suppressors by inhibiting tumor cell proliferation through regulation of IGF‐1 signaling. Another in vivo experiment showed that soluble Klotho possesses greater inhibitory effects on tumor cell growth than full‐length membrane Klotho.^(^ ^97^ ^)^ Therefore, through attenuation of TGFβ1‐, Wnt‐, and IGF1‐signaling pathways, Klotho also inhibits tumorigenesis. The promoter proximal region of the Klotho gene is reported to be hypermethylated in cancer, and transgenic overexpression or introduction of Klotho protein is observed to retard tumor growth in several animal models.

With respect to the antifibrotic qualities of Klotho, high concentrations of extracellular phosphate are toxic to cells, and impaired urinary phosphate excretion increases serum phosphate levels to induce a premature‐aging phenotype. Urinary phosphate levels are increased by dietary phosphate overload and might induce tubular injury and interstitial fibrosis. Extracellular phosphate exerts its cytotoxic effects by forming insoluble nanoparticles with calcium and fetuin‐A. These nanoparticles are referred to as calciprotein particles and are capable of inducing various cellular responses, including the osteogenic transformation of vascular smooth muscle cells and cell death of vascular endothelial cells and renal tubular epithelial cells. Calciprotein particles can be detected in the serum of animal models of kidney disease and in patients with chronic kidney disease (CKD) and probably contribute to the pathogenesis of CKD. This important insight provides a mechanism whereby Klotho, by preventing hyperphosphatemia, protects the vascular and renal systems, thereby prolonging lifespan. In addition, 1,25(OH)_2_D is thought to cooperate with Klotho in retarding vascular calcification by the induction of OPN (Fig. S1 *C*), a powerful antimineralization factor.^(^ ^60^ ^)^ Ironically, the two renal hormones that are deficient in patients with chronic renal failure because of renal mass loss, namely 1,25(OH)_2_D and Klotho, are two vital effectors of renal and vascular health, suggesting a strategy for the prevention and treatment of vascular disease, as well as CKD.

### It is controversial whether Klotho affects insulin secretion and sensitivity

It has been observed that Klotho increases the plasma membrane retention of TRPV2, leading to enhanced glucose‐triggered insulin secretion from pancreatic β cells.^(^ ^98^ ^)^ Vitamin D has long been known to promote insulin secretion,^(^ ^99^ ^)^ meaning that insulin release is yet another example of the dual beneficial effects of 1,25(OH)_2_D and Klotho. *Klotho* KO mice exhibit less energy storage and expenditure compared with WT mice,^(^ ^100^ ^)^ as well as attenuated insulin production and enhanced insulin sensitivity.^(^ ^101^, ^102^ ^)^ However, Anour and colleagues^(^ ^103^ ^)^ demonstrated that Klotho lacks a vitamin D‐independent physiological role in glucose homeostasis, bone turnover, and steady‐state PTH secretion, in vivo, casting doubt on Klotho alone as a bona fide regulator of any of these phenomena. In a sense, this original research by the Erben group^(^ ^103^ ^)^ was prescient and consistent with the recent opinion piece^(^ ^50^ ^)^ that “Klotho's effects on mineral homeostasis are fibroblast growth factor‐23 dependent.” Are all Klotho actions dependent upon FGFR1 signal transduction initiated by FGF23? This is the provocative question regarding Klotho in 2020! Because FGFR1 is almost universally expressed, are all of the influences of Klotho on aging actually promulgated by FGF23 signaling? In an opposing theory, Dalton and colleagues^(^ ^78^ ^)^ propose that soluble Klotho targets GM1 and GM3 sialogangliosides clustered in membrane lipid rafts which act as a Klotho “receptor” that signals control of growth factor functions. Regardless of the mechanism by which Klotho functions, the many consequences of Klotho activity, such as antioxidation, antifibrosis, antimalignancy, anticalcium transients, and antiphosphatemia, clearly collectively contribute to the antiaging potential of Klotho. Based on all these observations, Klotho can be considered a 1,25(OH)_2_D/VDR‐induced, organ‐protection hormone that promotes healthful aging by delaying chronic diseases, even if exclusively through beneficial signaling via FGFR1.

Accordingly, both FGF23 and Klotho have recently been implicated in maintaining male reproductive function.^(^ ^104^ ^)^ Indeed, Hansen and colleagues,^(^ ^104^ ^)^ employing mice null for either FGF23 or Klotho, found that global loss of either FGF23 or Klotho compromised testicular weight and reduced sperm count as well as motility, whereas FGF23 enhances testicular weight in WT mice. However, germ‐cell–specific knockout of Klotho elicited neither decreased sperm count nor mobility, although fewer pregnancies and Klotho heterozygous pups occurred in this group that was characterized by overexpression of testicular trpv5 and npt2b,^(^ ^104^ ^)^ indicating that testicular calcium and phosphate exchange play a role in the actions of FGF23 and Klotho in gonads, as they do in the functions of FGF23 and Klotho in kidney.

### Klotho supports synaptic functioning and protects the central nervous system

In recent years, interest has arisen in the potential role of Klotho in the brain, including its possible protection of the central nervous system (CNS) and prevention of neurological diseases such as depression and the decline in cognitive function associated with aging.^(^ ^105^, ^106^ ^)^ Indeed, high levels of Klotho originating in the choroid plexus are postulated to function as a gatekeeper at the interface between the brain and immune system in the choroid plexus.^(^ ^107^ ^)^ Klotho depletion in aging or disease may weaken this barrier and promote immunomediated neuropathogenesis. Experimental depletion of Klotho from the choroid plexus enhanced microglial activation in the hippocampus after peripheral injection of mice with lipopolysaccharide. In primary cultures, Klotho suppressed thioredoxin‐interacting protein‐dependent activation of the NLRP3 inflammasome in macrophages by enhancing FGF23 signaling.^(^ ^107^ ^)^ Finally, peripheral delivery of an α‐Klotho fragment acutely enhanced cognition and neural resilience in young, aging, and disease‐model mice by inducing GluN2B cleavage and increasing NMDAR‐dependent synaptic plasticity.^(^ ^108^ ^)^ Thus, as 1,25(OH)_2_D is hypothesized to affect the CNS, Klotho induction by 1,25(OH)_2_D/VDR appears to be able to accomplish similar enhancement of synaptic functions—leading to the proposal that part of the benefits to brain health afforded by 1,25(OH)_2_D are mediated by its primary induction of Klotho, analogous to the known improvements in renal and vascular health promulgated by 1,25(OH)_2_D/VDR action to boost Klotho.

### Summary of the significant actions of Klotho that are potentially antipathogenic

In summary, the primary genomic induction of renal Klotho by 1,25(OH)_2_D/VDR–RXR leads to the following extensive list of biological consequences that benefit the entire organism because Klotho: (i) acts as a renal coreceptor for FGF23 in binding to FGFR isoforms that signal feedback control of hyperphosphatemia and excessive 1,25(OH)_2_D levels, both of which can lead to vascular calcification via osteogenic transformation of vascular smooth muscle cells and vascular endothelial cell death, as well as to renal tubular epithelial cell death resulting in chronic kidney disease; (ii) exerts antioxidative effects and controls intracellular calcium currents to protect tissues from the ravages of aging; (iii) blunts TGFβ1 and Wnt signaling to prevent renal and cardiac fibrotic disease, as well as tumorigenesis; (iv) acts in the CNS to enhance synaptic functions that mediate plasticity to support neural resiliency and cognition, as well as to protect against immunomediated degenerative neuropathogenesis. Although Klotho and its vitamin D hormone inducer may only play a small part in healthful aging, the sheer number of pathways impacted by this duo of renal hormones leads one to suspect that their pleiotropic actions are certainly a crucial factor in the quality and quantity of life. Because potentiation of FGF23/FGFR1 signaling is the only proven function of Klotho, the next section of this review addresses the induction of the FGF23 ligand by the 1,25(OH)_2_D renal hormone.

## Model for Secondary Regulation of Fibroblast Growth Factor‐23 by 1,25(OH)_2_D/VDR

### Primary and secondary regulation of gene expression by 1,25(OH)_2_D/VDR‐RXR

All biologic actions of 1,25(OH)_2_D/VDR appear to be mediated by association of liganded VDR–RXR with VDREs in target genes. In addition to the case of Klotho, discussed in detail above, other principal examples of this primary mechanism, established via ChIP‐seq results that prove the existence of this receptor protein–DNA molecular interaction, include 1,25(OH)_2_D/VDR‐mediated regulation of the expression of the following genes: (i) *cyp24a1*,^(^ ^109^ ^)^ (ii) *TRPV6*,^(^ ^110^ ^)^ (iii) *mmp13*,^(^ ^76^ ^)^ (iv) RANKL (*TNFSF11*),^(^ ^55^ ^)^ (v) osteocalcin (*BGLAP*),^(^ ^111^ ^)^ (vi) OPN *(spp1)*,^(^ ^112^ ^)^ (vii) *LRP5*,^(^ ^113^ ^)^ (viii) *CBS*,^(^ ^114^ ^)^ and (ix) leptin (*ob*).^(^ ^115^ ^)^ Thus, expression of all of the afore‐cited genes is governed in a primary fashion by 1,25(OH)_2_D/VDR–RXR. However, there exist numerous additional genes encoding functional proteins for which no VDREs have been identified, yet their expression is modulated by vitamin D. This category of genes is likely regulated secondarily, employing mediator transcription factor proteins. Consequently, 1,25(OH)_2_D‐directed stimulation in this class of vitamin D‐modulated genes is sensitive to inhibition by cycloheximide (an inhibitor of protein synthesis), which is a signature property of secondary regulation of gene expression. One such gene is FGF23, the bone‐derived phosphaturic peptide hormone that is induced by 1,25(OH)_2_D in a cycloheximide‐sensitive fashion,^(^ ^48^ ^)^ and that is apparently devoid of proven VDREs.^(^ ^116^ ^)^

### FGF2‐ and 1,25(OH)_2_D‐stimulated expression of the gene‐encoding FGF23

Bioinformatic analysis of the *FGF23* gene reveals that the immediate proximal promoter sequence (adjacent to the TATA‐box) harbors a conserved cAMP responsive element (CRE) as well as conserved NFAT (nuclear factor of activated T cells) and c‐ets1 elements, the former two of which have been shown by point mutation to be required for induction of FGF23 by FGF2.^(^ ^117^ ^)^ Therefore, a major regulator of FGF23 is FGF2 and its receptor, FGFR1, the nuclear fragment of which integrates gene regulation of ontogeny.^(^ ^118^ ^)^ With respect to vitamin D control of FGF23 gene expression, the induction of FGF23 by 1,25(OH)_2_D in mouse bone in vivo was first reported by Kolek and colleagues.^(^ ^48^ ^)^ In UMR‐106 osteosarcoma cells, FGF23 mRNA upregulation was shown to be 1,25(OH)_2_D‐dose dependent, occurring as early as 4 hours and peaking at 24 hours after exposure to the vitamin D hormone; but the effect was abrogated in the presence of cycloheximide, indicating dependence on an intermediary transfactor protein(s).^(^ ^48^ ^)^ Subsequent experiments performed by Saini and colleagues^(^ ^119^ ^)^ verified the dramatic induction of FGF23 in UMR‐106 osteosarcoma cells and revealed that nonmalignant, normal‐outgrowth cells from rat calvariae responded to 1,25(OH)_2_D by displaying over a fivefold enhancement of FGF23 mRNA when treated with 10nM 1,25(OH)_2_D. Moreover, leptin was reported to enhance 1,25(OH)_2_D‐dependent FGF23 induction whereas IL‐6 elicited suppression, intimating that other transfactors impacted FGF23 expression in the presence of 1,25(OH)_2_D.^(^ ^119^ ^)^ One such factor, c‐ets1, for which conserved responsive elements occur in the FGF23 gene, is induced by 1,25(OH)_2_D,^(119^ ^)^ and therefore emerges as a candidate mediator of FGF23 induction by 1,25(OH)_2_D. Next, Kaneko and colleagues^(^ ^120^ ^)^ dissected the mouse FGF23 promoter proximal sequence cloned by Ito and colleagues,^(^ ^121^ ^)^ discovering via truncation analysis in transfected human myelogenous leukemia K562 cells that the 1,25(OH)_2_D‐responsive region lies between −400 and −200 bp in vitro. Furthermore, Kaneko and colleagues^(120)^ noted that this region contains a duo of conserved elements, namely a c‐ets1‐like and a putative nurr1 cis sequence, either of which when point mutated, eliminated transcriptional activation by 1,25(OH)_2_D. Next, Onal and colleagues^(^ ^122^ ^)^ employed CRISPR‐Cas9 technology to obtain evidence for a distal enhancer in the mouse *fgf23* gene at −16 kb that mediates both inflammation‐ and PTH‐induced *fgf23* expression. Recently, utilizing this same unbiased in vivo method, Lee and colleagues^(^ ^123^ ^)^ localized 1,25(OH)_2_D responsiveness in the mouse to a sequence extending from −115 to −4110 bp upstream of the *FGF23* transcription start site. They also reported that this 4 kb promoter proximal region mediates *fgf23* response to high phosphate challenge and contains a second LPS/inflammation‐targeted cis‐element(s). One goal in the current review is to further define the 1,25(OH)_2_D‐responsive region of the mouse *fgf23* gene by re‐examining the proximal promoter region of the gene, and to develop a novel model of secondary regulation that reveals a heretofore unappreciated putative mediator(s) of this secondary response to 1,25(OH)_2_D/VDR–RXR.

### Induction of cyp24a1, fgf23, OPN, and nurr1 mRNAs by 1,25(OH)_2_D in UMR‐106

To gain further insight into the signal transduction pathway(s) employed by 1,25(OH)_2_D/VDR to induce FGF23, the kinetics of FGF23 mRNA enhancement by 1,25(OH)_2_D in UMR‐106 osteosarcoma cells (a cell line that exhibits osteocyte‐like character) was compared with that of other vitamin D‐induced genes such as *cyp24a1* (Fig. S1 *A*). Because it is a primary target for 1,25(OH)_2_D/VDR–RXR, cyp24a1 mRNA displays a rapid induction by 10nM 1,25(OH)_2_D, first significantly and appreciably (200‐fold) upregulated 2 hours after 1,25(OH)_2_D treatment and peaking 8 hours subsequent to hormone dosing, after which it decays to only a slight (14‐fold) enhancement by 48 hours. This relatively rapid mRNA time course can be considered the signature of a gene that is induced in a primary fashion by 1,25(OH)_2_D/VDR–RXR. As is evident in Fig. S1 *B*, FGF23 mRNA is much more slowly induced by exposure to 10nM 1,25(OH)_2_D, first appearing upregulated statistically significantly 2 hours post‐1,25(OH)_2_D, but very modestly (ninefold) and escalating minimally to 17‐fold at 4 hours of hormone treatment. FGF23 mRNA is not induced impressively (110‐fold) until 8 hours after 1,25(OH)_2_D treatment, at which point cyp24a1 mRNA enhancement has already maximized. Thus, there exists a clear temporal lag in the 1,25(OH)_2_D response of FGF23 compared with that of a primary gene (cyp24a1) in the same UMR‐106 target cells. As depicted in Fig. S1 *B*, FGF23 mRNA peaks broadly at approximately 500‐fold induction between 12 to 24 hours after hormone dosing, with the mean time of 18 hours being over twice as long as that of the appearance of the peak cyp24a1 response to 1,25(OH)_2_D (Fig. S1 *A*). FGF23 mRNA then declines much more slowly, maintaining a level of 89‐fold induction at 48 hours post‐1,25(OH)_2_D exposure. Therefore, the supplemental data in Fig. S1 *A*,*B* are consistent with FGF23 being induced by 1,25(OH)_2_D via a secondary, cycloheximide‐sensitive mechanism.

Somewhat surprising is the observation that the OPN gene, while endowed with a bona fide VDRE similar to cyp24a1, has an “intermediate” time course of induction. In Fig. S1 *C*, OPN *(spp1)* mRNA is statistically significantly induced at 2 hours (threefold), 4 hours (sixfold), and 8 hours (28‐fold) after 10nM 1,25(OH)_2_D treatment, with a sharp peak (91‐fold) 12 hours after hormone dosing, more reminiscent of the initial rate of FGF23 induction by 1,25(OH)_2_D rather than the kinetics of cyp24a1 mRNA enrichment. However, this OPN time course of 1,25(OH)_2_D response with a peak induction at 12 hours post‐1,25(OH)_2_D falls temporally between that for cyp24a1 (8 hours peak) and fgf23 (18 hours peak), instead of aligning with that for either the established primary or secondary mechanisms. That the OPN response to 1,25(OH)_2_D is more rapid than that of FGF23 is consistent with VDRE(s) in the *spp1* gene, but the hybrid nature of OPN's temporal induction suggests that a mediator protein may be required for full stimulation of *spp1* gene expression by 1,25(OH)_2_D. This observation implies (but does not prove) that nurr1, which we originally hypothesized,^(^ ^116^ ^)^ at least in part, mediates induction of FGF23 by 1,25(OH)_2_D, might comprise a significant secondary supplement to 1,25(OH)_2_D in the stimulation of OPN transcription, especially considering that nurr1 alone is fully capable of inducing bone‐expressed genes such as OPN^(^ ^124^ ^)^ and osteocalcin,^(^ ^125^ ^)^ and that 1,25(OH)_2_D synergistically enhances OPN mRNA when added with nurr1.^(^ ^124^ ^)^ As is depicted in Fig. S1 *D*, when UMR‐106 cells are dosed with the supraphysiologic concentration of 100nM 1,25(OH)_2_D, nurr1 mRNA is induced extremely rapidly, being statistically significantly upregulated (3.4‐fold) 30 minutes after sterol hormone exposure and peaking between 30 minutes and 1 hour of treatment. However, nurr1 mRNA induction decays to baseline levels by 12 hours post‐1,25(OH)_2_D treatment, despite the fact that the dose of 1,25(OH)_2_D was elevated 10‐fold to 100nM in this experiment only (Fig. S1 *D*). Meir and colleagues^(^ ^126^ ^)^ have similarly reported that nurr1 is induced very rapidly by 1,25(OH)_2_D in UMR‐106 cells with a quick (1 hour) spike in mRNA. Thus, nurr1 appears to satisfy one criterion for a 1,25(OH)_2_D‐dependent, primary mediator of FGF23 induction, as well as a secondary enhancer of OPN stimulation, namely that it is upregulated temporally prior to the ultimate (secondary) gene products. Finally, as documented in Fig. 4*B*, the proximal promoter region of the mouse *spp1* gene possesses a classic VDRE that is flanked on the 5′‐side by a runx2 site and on the 3′‐side by a c/ebp‐β element, and this collection of elements is bisected by a cluster of two nurr1 sites and a c‐ets1 element. Therefore, it is not unreasonable to assume that OPN control is exercised by multiple inputs, in addition to the 1,25(OH)_2_D ligand, which employs transfactor mediators that signal transcriptional activation of the *spp1*‐enhancer module anchored by the VDRE.

**Fig 5:**
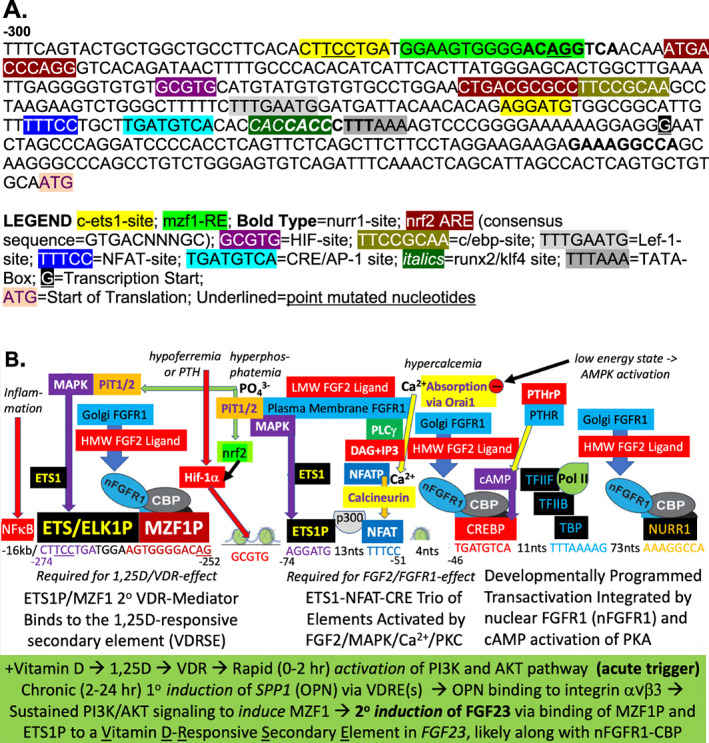
(*A*) Promoter proximal DNA sequence of the mouse *Fgf23* gene, and (*B*) a model for the regulation of mouse FGF23 by 1,25(OH)_2_D and multiple inputs (hypoferremia, PTH, hyperphosphatemia, hypercalcemia, and low‐energy state) mediated by various transcription factors. HMW= high molecular weight; LMW = low molecular weight; NFAT = nuclear factor of activated T cells; OPN = osteopontin; VDR = vitamin D receptor; VDREs = vitamin D responsive elements.

However, with respect to possible secondary regulation of FGF23 by nurr1, the putative nurr1 element identified by our group,^(^ ^120^ ^)^ between −256 bp and −248 bp in the mouse fgf23 gene (Fig. 5*A*), has drawbacks in that its **G**A**C**AGGTCA sequence differs from the nurr1 consensus because the #1 and #3 positions are guanine and cytidine, respectively. In the original identification of the NURR1/NGFIB responsive element (NBRE), Wilson and colleagues^(^ ^127^ ^)^ reported that **T**A**T**AGGTCA still binds NURR1, but not as strongly as AAAAGGTCA (the consensus). This suggests that the #1 and #3 positions in the NBRE may be “flexible,” although Murphey and colleagues^(^ ^128^ ^)^ showed that though the #1 base is “flexible,” the #3 position must be an adenine for NURR1 binding to occur. This later finding caused us to re‐examine whether the NURR1 element we originally identified^(^ ^120^ ^)^ in fact attracted a different transfactor. We also noted that this sequence in the mouse fgf23 proximal promoter region overlapped, and possibly conflicted sterically in terms of putatively bound transfactors, with a conserved upstream sequence identified as an MZF1 cis element (see Fig. 5*A*). Because of these concerns for the validity of the “NURR1” element, combined with compelling precedence detailed below that MZF1 is the likely transfactor that actually mediates, along with c‐ETS1, the secondary response of FGF23 mRNA to 1,25(OH)_2_D/VDR, we offer an updated mechanism for how 1,25(OH)_2_D and other regulators actually induce *fgf23* gene expression.

### MZF1/ETS1 proposed as transcriptional mediators of fgf23 induction by 1,25(OH)_2_D

The genesis of our new model is that site‐directed mutation of either the MZF1 or c‐ETS1 target sequence in the mouse *fgf23* gene, between nucleotides −274 and −250 bp, as shown in Fig. 5*A*,*B* (mutated nucleotides underlined), abrogates the 1,25(OH)_2_D responsiveness of *fgf23*.^(^ ^116^ ^)^ Our interest in MZF1 and c‐ETS1 as candidate intermediaries in the induction of FGF23 by 1,25(OH)_2_D was fueled by the report from Yue and colleagues^(^ ^129^ ^)^ that a protein–protein complex of MZF1 and ELK1 (a member of the Ets family of transcription factors) binds to the PKCα proximal promoter region, specifically to the sequence aggaTGGGGAaggggCTTCCTgct (MZF1‐ and ELK‐responsive elements in uppercase lettering, respectively) in hepatocarcinoma. Therefore, PKCα is induced through MZF1/ELK1 mediator signaling.^(^ ^129^ ^)^ Also, PKCα is known to be required for OPN upregulation in osteoblasts exposed to excess inorganic phosphate.^(^ ^130^ ^)^ More striking is that the responsive nucleotide sequence in PKCα (defined above) dramatically resembles the 1,25(OH)_2_D‐responsive motif in the mouse *fgf23* gene (Fig. 5*A*) in that it possesses identical MZF1 (TGGGGA) and ETS1 (CTTCCT) juxtaposed core sequences, although they are 5′‐ to 3′‐switched between the two genes. This constitutes indirect evidence that the target enhancer sequence for 1,25(OH)_2_D identified by site‐directed mutagenesis in the mouse *fgf23* gene highlighted in Fig. 5 (**CTTCCT**GATGGAAG**TGGGGA**CAGG**)** actually responds to MZF1/ETS1 as a secondary mediator(s) of 1,25(OH)_2_D/VDR action in bone cells. Moreover, MZF1 is expressed in human osteoblasts and regulates the level of mRNA encoding the bone‐expressed gene, N‐cadherin.^(^ ^131^ ^)^ A MZF1‐binding motif, aagggggTGGGGAggggaggg, is present in the promoter proximal region of the human N‐cadherin gene and has been shown to cooperate with a nearby SP‐1 element, with the SP‐1/MZF1 duo of transfactors acting to stimulate N‐cadherin expression.^(^ ^131^ ^)^ Importantly, if SP‐1 is substituted by SP‐3, expression of N‐cadherin is repressed, indicating that the composition of the MZF1 partner determines the direction whereby MZF1 controls transcription. These revelations in the literature concerning the MZF1 motif provide scientific underpinning for our novel hypothesis that c‐ETS1 and MZF1 constitute dual transfactor mediators of the *fgf23* mRNA response to 1,25(OH)_2_D/VDR. Finally, because point mutation of either of these two motifs in the mouse *fgf23* promoter proximal domain ablates 1,25(OH)_2_D responsiveness,^(^ ^120^ ^)^ we propose herein that at least mzf1 comprises a cycloheximide‐sensitive mediator of 1,25(OH)_2_D's secondary action to induce *fgf23* mRNA.

The present hypothesis that mzf1 is the “first‐responder” in the induction of fgf23 by 1,25(OH)_2_D is also supported by research results from Reiner's group.^(^ ^132^, ^133^ ^)^ They have studied myeloid cell differentiation in response to 1,25(OH)_2_D and observed that *CD11b* and *CD14*, two monocytic differentiation markers, are dependent on MZF1 as an intermediary transfactor for their induction by 1,25(OH)_2_D/VDR.^(^ ^133^ ^)^ The proximal promoter regions of both genes do not contain classical VDREs, but possess consensus MZF1‐binding sites, namely AG**TGGGGA**, and these sites are occupied by MZF1 protein after treatment of myeloid cells with 1,25(OH)_2_D.^(^ ^133^ ^)^ In their earlier study, the Reiner team reported that the mechanism of action of 1,25(OH)_2_D in myeloid differentiation is mediated by a VDR‐PI3K signaling complex rather than via the traditional action of VDR–RXR at the genomic level.^(^ ^132^ ^)^ The evidence for this nongenomic mechanism consisted of the rapidity (5–30 minutes) of the PI3K activation response to 1,25(OH)_2_D, and its sensitivity to being attenuated by PI3K inhibitors, antisense oligonucleotides encoding the mRNA for the p110 catalytic subunit of PI3K, and a dominant negative mutant of PI3K.^(^ ^132^ ^)^ These data indicate that PI3K is essential for 1,25(OH)_2_D to signal the induction of *CD11b* and *CD14* in differentiating myeloid cells and are consistent with our hypothesis that MZF1 constitutes an intermediary transcription factor in 1,25(OH)_2_D/VDR‐induced expression of genes such as *fgf23*. Moreover, Hmama and colleagues^(^ ^132^ ^)^ concluded, based upon rapid activation (20 minutes) of PI3K by 1,25(OH)_2_D and coimmunoprecipitation of VDR with PI3K, that 1,25(OH)_2_D‐liganded VDR is directly activating PI3K.

### Nongenomic actions of 1,25(OH)_2_D/VDR initiate acute effects via PI3K signaling

There is considerable published evidence that 1,25(OH)_2_D acts nongenomically, either in combination with extranuclear VDR or putative plasma membrane 1,25(OH)_2_D‐binding proteins, to elicit rapid cellular effects such as activation of multiple protein kinases and phospholipases, as well as phosphatidylinositol‐3 kinase (PI3K) and p21ras.^(^ ^134^ ^)^ 1,25(OH)_2_D also appears to quickly generate second messengers such as Ca^2+^, cyclic AMP, fatty acids, and 3‐phosphoinositides, in concert with the activation of protein kinase A, src kinase, mitogen‐activated protein kinases, protein kinase C, and Ca^2+^‐calmodulin kinase II.^(^ ^134^ ^)^ Anthony W. Norman was a champion of nongenomic, “fast action” of 1,25(OH)_2_D, and made its study a major thrust in the latter stages of his career,^(^ ^66^, ^135^, ^136^, ^137^ ^)^ after his laboratory pioneered the genomic mechanism of action as described at the beginning of this review. Specifically, Vertino and colleagues^(^ ^136^ ^)^ showed that a ligand‐binding domain‐only construct of VDR functioned in osteocytes to mediate the antiapoptotic influence of 1,25(OH)_2_D, and Zhang and Zanello^(^ ^137^ ^)^ reported that 1,25(OH)_2_D very rapidly activated PI3K/AKT antiapoptotic signaling in ROS‐17/2.8 osteoblasts. Therefore, it is of particular note that rapid nongenomic activation of PI3K may be seminal to the induction of FGF23 by 1,25(OH)_2_D/VDR through the MZF1 transcriptional mediator as discussed above. Moreover, by rapidly activating MAP kinases such as erk5,^(^ ^134^ ^)^ which employs c‐ETS1 as a substrate, nongenomic 1,25(OH)_2_D function could also contribute to *fgf23* induction through phosphorylation of c‐ETS1 to c‐ETS1‐P, activating it to enter the nucleus and bind cooperatively with MZF1 to complete the duo of transintermediaries we propose herein are the key to the secondary induction of *fgf23* by 1,25(OH)_2_D/VDR. In this fashion, rapid nongenomic effects of 1,25(OH)_2_D may constitute the initial molecular mechanism whereby 1,25(OH)_2_D/VDR, without the benefit of its RXR heteropartner, sparks the secondary induction of *fgf23* via the MZF1/c‐ETS1‐P intermediaries, and this may be the first example of a gene induced by 1,25(OH)_2_D that “integrates” both genomic and nongenomic modes of vitamin D action.

In fact, we suggest that the mechanism put forth by Hmama and colleagues^(^ ^132^ ^)^ and reinstituted by Moeenrezakhanlou and colleagues^(^ ^133^ ^)^ may not be the complete molecular explanation for how VDR mediates induction of *CD11b* and *CD14* and concomitant myeloid cell differentiation. Because myeloid cells also express OPN that is induced in a primary and genomic manner by 1,25(OH)_2_D,^(^ ^138^ ^)^ we herein put forth OPN as a candidate sustainer of the acute and likely transient [peaking at 20 minutes post‐1,25(OH)_2_D treatment] effect of 1,25(OH)_2_D to activate PI3K by chronically activating PI3K signaling to induce MZF1. By virtue of OPN's dramatic primary induction in response to 1,25(OH)_2_D/VDR, we propose OPN is the protein that drives MZF1 enhancement for the chronic “long‐haul.” What is the evidence that OPN is capable of upregulating MZF1?

### Osteopontin controls bone mineral deposition in conjunction with FGF23

Osteopontin, which is a benchmark 1,25(OH)_2_D/VDR‐induced protein in bone, is a novel upstream protein that is a potent and rapid stimulator of *MZF1* expression, specifically in human mesenchymal stem cells, where subsequent MZF1‐driven TGFβ signaling causes these cells to differentiate and adopt a cancer‐associated fibroblast phenotype.^(^ ^139^ ^)^ By engaging αvβ3 integrin on the cell surface, OPN activates PI3K/AKT signaling to influence numerous pathways, including those involving mTOR and β‐catenin.^(^ ^140^ ^)^ Interestingly, mTOR expression is downregulated by MZF1 as evidenced by the significant suppression of *mtor* promoter activity upon MZF1 overexpression in BALB/c mice.^(^ ^141^ ^)^ MZF1 overexpression also reduced MTOR expression in both fibroblasts and mouse plasmacytoma cells, with ChIP‐PCR confirming that MZF1 binds to the core cis‐element TGGGGA located at −6/−1 in the *mtor* promoter region.^(^ ^141^ ^)^

How does sustained MZF1 enhancement come about in cells, including osteoblasts? Although it remains possible that MZF1 is primarily/directly induced by 1,25(OH)_2_D via an uncharacterized VDRE, we propose, as introduced above, that MZF1 appears after 1,25(OH)_2_D treatment through the intermediary gene product, OPN, which is a well‐established direct 1,25(OH)_2_D/VDR target.^(^ ^112^ ^)^ Figure S1 *C* illustrates that *spp1* is highly and rapidly induced by 1,25(OH)_2_D in UMR‐106 cells. Because OPN is a bona fide stimulator of MZF1 expression,^(^ ^139^ ^)^ we herein hypothesize that OPN is a second cycloheximide‐sensitive protein that is the key to understanding how 1,25(OH)_2_D induces *fgf23* in a secondary fashion and maintains this induction over the long‐term. Thus, through PI3K/AKT signaling, OPN upregulates MZF1, likely via an AKT pathway to elicit the occupation of c‐myb and AP4 elements that have been shown to be functional within the MZF1 proximal promoter region.^(^ ^142^ ^)^ Notably, Chen and colleagues^(^ ^142^ ^)^ reported that enhanced MZF1 expression suppresses prostate tumor cell growth by promoting ferroportin‐driven iron egress as a result of MZF1 induction of ferroportin. The MZF1/ferroportin axis is likely tied to FGF23 because iron is a proven regulator of FGF23 through HIF1α.^(^ ^143^ ^)^ In a sense, this observation “closes the signaling circle” and further implicates MZF1 as a crucial FGF23‐modulating transcription factor. It also solidifies the position of SPP1 as a driver gene in bone and bone mineral homeostasis because the physiologic role of FGF23 is to limit bone mineralization, and FGF23 executes this task in many ways. Importantly, FGF23 retards renal phosphate reabsorption to mitigate mineralization and reduces circulating 1,25(OH)_2_D to lessen calcium and phosphate absorption, but it also requires the cooperation of agents such as the OPN sibling protein to act extracellularly in bone to blunt the crystallization process locally. Therefore, OPN does the local work of governing bone mineral accretion, but also apparently ensures that sufficient FGF23 is available to protect the skeleton from excessive calcification. We assert that OPN, like its partner growth factor elaborated by bone, FGF23, should be considered a novel bone hormone. After induction by 1,25(OH)_2_D/VDR, OPN acts as a sentinel for skeletal mineralization in several modes. It therefore makes biological sense that OPN should be the sustaining intermediary between 1,25(OH)_2_D/VDR and FGF23, linking a rapid nongenomic PI3K activation by 1,25(OH)_2_D to a continuing response of PI3K signaling driven by autocrine OPN binding to integrin αvβ3.

The conclusion is that the results published by Reiner's group^(^ ^132^, ^133^ ^)^ provide a compelling analogy to the present hypothesis that 1,25(OH)_2_D/VDR acts secondarily to induce FGF23 in osteocytes via the following sequence of events: (i) Rapid (0–1 hour) nongenomic function of 1,25(OH)_2_D/VDR to activate PI3K signaling^(^ ^136^, ^137^ ^)^ that produces a “starter” quantity of MZF1 to “prime the pump”; (ii) VDRE‐mediated enhancement of OPN (1–12 hours) in a primary genomic response to 1,25(OH)_2_D/VDR; (iii) OPN binding to αvβ3 integrin to signal protracted PI3K/AKT stimulation; (iv) PI3K/AKT signaling the sustained induction of MZF1; (v) MZF1, along with a partner transcription factor member of the c‐ETS family, binding to cognate cis elements in the proximal promoter region (−252 to −274 bp) of the murine *fgf23* gene; and (vi) secondary transcriptional activation of *fgf23* gene expression through the recruitment of coactivator protein(s). The fact that this analogy arose from studies in myeloid cells is reminiscent of the original observation of Ito and colleagues^(^ ^121^ ^)^ that human myelogenous leukemia K562 cells were one of the few cell types that expressed significant levels of FGF23 mRNA, most likely because of the rich background of MZF1 that occurs in myeloid leukemia cells.

### FGF23 gene expression is regulated by multiple inputs beyond 1,25(OH)_2_D and FGF2

Figure 5*A* presents the proximal promoter sequence of the *fgf23* gene in the mouse. Apparent are numerous transcription factor cis‐element motifs conserved across species, including the following sites: (i) TATA‐box, (ii) CRE/AP‐1, (iii) NFAT, (iv) c‐ets1, (v) Lef‐1, (vi) c/ebp, (vii) nrf2, (viii) HIF1α, (ix) nurr1, and (x) MZF1. Although any of these sites could potentially contribute to the secondary response of *fgf23* expression to 1,25(OH)_2_D/VDR by docking a key transfactor that is influenced by 1,25(OH)_2_D, we have herein narrowed‐down the potential sequence that likely comprises the vitamin D responsive secondary element (VDRSE) to nucleotides −252 to −274, a sequence that contains consensus MZF1 and c‐ETS1 cis elements. To summarize and place vitamin D control of *fgf23* gene expression in the general context of FGF23 developmental and physiological modulation, Fig. 5*B* is presented as a model that includes the 1,25(OH)_2_D‐dependent segment of the mouse *fgf23* gene (*left*), with both putative transfactors, namely c‐ETS1 and MZF1, bound to their cognate elements. In Fig. 5*B*, the −252 bp to −274 bp sequence in *fgf23* is designated as a 1,25(OH)_2_D‐responsive secondary element to differentiate it from the traditional 15 bp sequence of the primary VDRE. We do not yet understand exactly how c‐ETS1 participates molecularly in the enhancement of *fgf23* expression by 1,25(OH)_2_D, other than it seems to be an obligatory partner to MZF1. Because c‐ets1 employs VDR as one of its many transcription factor cohorts,^(^ ^144^ ^)^ it is not out of the realm of possibility that c‐ets1 could attract unliganded VDR to the VDRSE as a “helper” coactivator.^(^ ^145^ ^)^ More likely, c‐ets1 cooperates with MZF1 in recruiting one or more of its documented alternative cointegrators.^(^ ^144^ ^)^ MZF1 is a transcription factor in the Kruppel family of zinc finger proteins that is expressed predominantly in myeloid progenitor cells. Indeed, MZF1 participates in controlling growth, differentiation, and apoptosis of progenitors, regulating transcription during differentiation along the myeloid lineage.^(^ ^146^ ^)^ Yet, in an important advance, Le Mee and colleagues^(^ ^131^ ^)^ discovered that MZF1 induced human N‐cadherin in osteoblasts, suggesting that MZF1 could be crucial in skeletal as well as myeloid cells. Notably, cadherins are transmembrane glycoproteins that guide calcium‐dependent cell–cell adhesion during morphogenesis and development. They also complex with catenins to govern a variety of processes including cell–cell adhesion, signal transduction, and control of gene transcription.^(^ ^147^ ^)^ Because control of FGF23 spans bone morphogenesis, development, and differentiated functions, it is intriguing that the cadherin–catenin interrelationship may be relevant to regulation of FGF23 via the MZF1 connection. Accordingly, it has been reported recently by Ko and colleagues^(148)^ that phosphorylation‐dependent stabilization of MZF1 upregulates N‐cadherin expression during protein kinase CK2‐mediated epithelial‐mesenchymal transition in human esophageal cancer cell lines TE2 and HCE4. MZF1 is phosphorylated by CK2 at serine 27 that activates the transcription factor for DNA‐responsive element association and stabilizes it to prevent degradation via proteolysis, thereby escalating MZF1 protein concentration and in turn amplifying transcription of N‐cadherin.^(^ ^148^ ^)^ In an amazing molecular connection between induction of FGF23 by 1,25(OH)_2_D/VDR and CK2‐catalyzed phosphorylation, VDR is known to be phosphorylated by CK2,^(43^ ^)^ and this posttranslational modification enhances the transactivation function of VDR,^(^ ^44^ ^)^ likely by stabilizing the receptor protein. Equally intriguing is the recognition of CK2, a protein kinase that participates in organ development, as a key biochemical player in *fgf23* expression during the ontogeny of bone development. We postulate that CK2 may comprise an endogenous switch that modulates gene expression during cell growth and differentiation.

It is likely that endocrine control of FGF23 occurs postnatally to ensure homeostasis, whereas ontogenic modulation of FGF23 takes place during development, triggered by the FGF2/FGFR1/nFGFR1 signal cascade being activated by the FGF2 growth factor and possibly PTHrP originating in chondrocytes to stimulate bone morphogenesis (Fig. 5*B*, *upper right*). As is the case for PTH, cAMP is the second messenger for PTHrP that functions through phospho‐CREB binding to the conserved CRE near the TATA‐box in the *FGF23* gene (Fig. 5*B*, *right center*). But as reported by Han and colleagues,^(^ ^117^ ^)^ for FGF2 to enhance FGF23 expression, other required second messengers are generated through low‐molecular‐weight (LMW) FGF2‐ligand binding to its plasma membrane FGFR1, such as diacylglycerol that activates PKC and inositol triphosphate (IP3)/Ca^2+^ to activate calcineurin‐catalyzed dephosphorylation of NFAT, translocating it into the nucleus to bind the NFAT element in DNA. Finally, included in the proposed model of *FGF23* gene regulation is the activation of MAP kinase (MAPK) when plasma membrane FGFR1 is liganded with LMW FGF2 (Fig. 5*B*, *center*). Phosphorylation of c‐ETS1 by MAPK is required for this transcription factor to associate with its cognate element in the *fgf23* proximal promoter sequence at −74 bp, thereby allowing it to cooperate with NFAT to stimulate *fgf23* transcription.^(^ ^117^ ^)^

### Mineral, endocrine, and stress regulators of FGF23: Mechanisms of action

For completeness of the model proposed in Fig. 5*B*, several other established effectors of FGF23 homeostasis are included, namely inflammation, hypoferremia, hyperphosphatemia, PTH, hypercalcemia, and energy state. Inflammation‐triggered induction of *fgf23* is initiated by LPS and cytokines such as TNF‐α and IL‐1β, with the response executed by NFκB (p65/p50) binding to the *fgf23* gene in the −16 kb region^(^ ^122^ ^)^ (Fig. 5*B* left). Hypoferremia and likely inflammation also enhance *fgf23* gene expression via HIF‐1α/HIF‐1β bound to one of several HIFREs in the *fgf23* gene, and one such conserved HIFRE (GCGTG) is present in the proximal promoter sequence of the mouse *fgf23* gene at −165 bp (Fig. 5*A*,*B*). PTH also functions to enhance HIF‐1α in osteoblasts, as Frey and colleagues^(^ ^149^ ^)^ reported that HIF‐1α exerts distinct developmental functions and acts as a negative regulator of bone formation. Mice lacking HIF‐1α in osteoblasts and osteocytes formed more bone in response to intermittent PTH, which is known to be an anabolic agonist. Also, exposure of primary mouse osteoblasts to PTH resulted in the rapid induction of HIF‐1α protein levels via a post‐transcriptional mechanism. Similarly, deferoxamine, an iron chelator that inhibits the activity of the prolyl‐hydroxylase enzymes that initiate the targeting of HIF‐1α subunits for proteasomal degradation, elicits rapid induction of HIF‐1α protein levels by simulating hypoferremia, a known stimulator of *fgf23* induction via HIF‐1α augmentation as described above. Therefore, as illustrated in Fig. 5*B* (*left center*), we propose that both PTH and hypoferremia act through HIF‐1α to induce *fgf23* during exposure of osteoblasts to stresses of iron depletion and PTH function to diminish osteoblast mineralization, allowing for enhanced bone resorption.

Phosphate control of FGF23 is fundamental to skeletal physiology and is quite complex in nature, with high‐phosphate rapidly inducing *fgf23* as well as other bone‐expressed genes including *dmp1*, *spp1*, and *tnfsf11*.^(^ ^123^ ^)^ Neither the molecular sensor for phosphate nor the intracellular signal transduction pathway that mediates its influence on *fgf23* expression has been identified to date, although Lee and colleagues ^(123)^ recently localized the phosphate effect on FGF23 in the mouse to a sequence extending from −115 to −4110 bp upstream of the FGF23 transcription start site. The proximal promoter region of this phosphate‐responsive stretch of nucleotides is depicted in Fig. 5*A*, but has yet to be further dissected experimentally. Thus, to complete the model in Fig. 5*B* for *fgf23* regulatory mechanisms propagated by phosphate, we can only speculate on the actual molecular mediators. PiT1 is induced by 1,25(OH)_2_D,^(^ ^150^ ^)^ signals the binding of extracellular phosphate via the ERK1/2 protein kinase cascade,^(^ ^151^ ^)^ and inhibition of phosphate uptake by PiT1 small hairpin silencing RNA in cultured vascular smooth muscle cells (VSMCs) blocks the expression of phosphate‐induced osteogenic differentiation markers such as runx2 and OPN.^(^ ^152^ ^)^ Therefore, we postulate herein that PiT1 is a reasonable candidate for a phosphate sensor/transporter role in osteoblasts and/or hypertrophic chondrocytes. Recently, Bon and colleagues^(^ ^153^ ^)^ reported that a heterodimer of high affinity sodium‐dependent phosphate transporters PiT1/*Slc20a1* and PiT2/*Slc20a2* determines phosphate sensing independently of phosphate influx. This raises the possibility that PiT1/2 could constitute the long‐sought phosphate sensor on bone cells. Evidence for the functional significance of PiT1 and PiT2 is provided by experiments in which ablation of PiT1 or PiT2 suppressed both phosphate‐dependent ERK1/2 (MAPK)‐catalyzed phosphorylation and subsequent induction of the mineralization inhibitors matrix Gla protein and OPN.^(^ ^153^ ^)^ Because nrf2 is one transcription factor that is upregulated in osteoblasts exposed to high‐phosphate levels,^(^ ^154^ ^)^ we put forward nrf2 as a potential candidate to mediate the effect of phosphate exposure on *fgf23* gene expression (Fig. 5*B*). Perhaps relevant is the identification of a conserved responsive element for nrf2 in the *HIF1*α gene,^(^ ^155^ ^)^ meaning that HIF1α could be the transcription factor that mediates the response of *fgf23* expression to high phosphate as well as to hypoferremia and inflammation. Other candidate mediators include RUNX2, which is linked to the proposed PiT1/2 phosphate sensor by the observation that knockdown of PiT1 abrogates *runx2* induction by high‐phosphate challenge.^(^ ^152^ ^)^ It is likely that other transcription factors participate in the intricate and critical regulation of FGF23 by phosphate, particularly because FGF23 is the new dominant phosphate homeostasis peptide hormone, which was previously known as “phosphatonin” (initially as a counterpoint to the naming of the rather weak calcium‐lowering hormone, calcitonin). Ironically, FGF23 is also an effective calcium‐lowering hormone by virtue of its ability to suppress the principal calcemic hormone, 1,25(OH)_2_D. Finally, as expected in an endocrine sense, calcium is also a modulator of FGF23, with induction of FGF23 by hypercalcemia (Fig. 5*B*). David and colleagues^(^ ^156^ ^)^ showed that calcium increases FGF23 in bone, both in vivo and in cultured cells, in vitro. We demonstrated that a 1 kb mouse FGF23 promoter‐reporter construct, transfected into MC3T3‐E1 osteoblast‐like cells, responds to a high calcium challenge with a statistically significant (twofold) enhancement of transcription.^(^ ^120^ ^)^ Recently, in a preliminary experiment (data not shown) utilizing transfection of MC3T3‐E1 cells, we observed that calcium stimulation of transcription exists in the first 200 bp of the proximal promoter sequence in the mouse *fgf23* gene. These data are consistent with the conclusion of Han and colleagues ^(117)^ that the NFAT transcription factor may mediate the response of *fgf23* gene expression to calcium through PLCγ, IP3, and intracellular activation of calcineurin by calcium (Fig. 5*B*). Finally, calcium entry via ORAI1 has recently been reported^(^ ^157^ ^)^ to be inhibited by AMPK activation in low‐energy states (Fig. 5*B*), which would be accompanied by low‐phosphate levels that are not exacerbated because *FGF23* expression is reduced in the absence of store‐operated calcium ion entry. Thus, in low‐energy situations, FGF23 synthesis and release are curtailed because calcineurin activation of NFAT is attenuated, thereby reducing *fgf23* gene expression. Clearly, the regulation of *FGF23* gene expression is managed by multiple inputs and all control systems must be in place and in perfect working order to prevent the occurrence of the many insidious chronic disorders that occur in conjunction with deranged levels of FGF23 and phosphate.

### Genomic/nongenomic 1,25(OH)_2_D actions via PI3K in bone, brain, and immune system

In conclusion, this review posits that whereas induction of Klotho by 1,25(OH)_2_D in renal cells appears to be accomplished by the traditional primary mechanism of liganded VDR‐RXR binding to functional VDREs in the region of the *KL* gene, the mechanisms that mediate regulation of FGF23 by 1,25(OH)_2_D/VDR are more complicated. Herein we recognize the role of 1,25(OH)_2_D‐liganded VDR in rapidly activating PI3K signaling to trigger certain bioactions in a mechanism that does not involve VDR‐DNA interaction, at least initially, specifically in osteoblasts^(^ ^137^ ^)^ and osteocytes,^(^ ^136^ ^)^ where inhibition of apoptosis was the 1,25(OH)_2_D action studied. In this nongenomic mechanism, 1,25(OH)_2_D/VDR quickly (5 minutes) associates with SRC‐kinase,^(^ ^136^ ^)^ possibly activating PI3K through phosphorylation of its regulatory subunit, with phospho‐PI3K signaling downstream activation of AKT, which in turn directs antiapoptosis of these bone cells. Importantly, this overall mechanism of antiapoptosis is sensitive to inhibition by actinomycin D,^(^ ^136^ ^)^ meaning that gene transcription of DNA to mRNA is still required for full biological responsiveness. Therefore, we conclude that to complete the rapid, nongenomic action of 1,25(OH)_2_D in osteoblasts/osteocytes, genomic effects of 1,25(OH)_2_D/VDR–RXR must ultimately come into play. To resolve this conundrum, in this review we present a novel paradigm that couples nongenomic and genomic actions of 1,25(OH)_2_D, specifically in the form of a new molecular explanation for how 1,25(OH)_2_D secondarily induces FGF23 in osteoblasts/osteocytes. The common denominator is PI3K and its ability to promulgate a vast number of signaling pathways that control cell survival, proliferation, growth, metabolism, and specialized cell functions such as neurotransmission. In the case of FGF23 induction in bone cells, we postulate that OPN is the crucial 1,25(OH)_2_D‐induced protein that sustains PI3K activation to generate a second induced protein, the MZF1 transcription factor, which in turn directly stimulates FGF23 transcription as detailed above in this section. In a sense, by genomically inducing OPN, 1,25(OH)_2_D is able to exert a protracted enhancement of FGF23 production by sustaining transient PI3K activation in osteoblasts that occurs via the nongenomic route.

The same independent, yet coupled set of events appears to transpire in the CNS where 1,25(OH)_2_D/VDR exerts both genomic and nongenomic influences.^(^ ^158^, ^159^ ^)^ Specifically, Sisley and colleagues^(^ ^160^ ^)^ demonstrated that 1,25(OH)_2_D acutely increases the firing frequency of paraventricular neurons through a PI3K‐dependent mechanism, while also inducing *Irs2* and *p85* in a genomic manner to amplify insulin signaling in hypothalamic cell culture, also via a PI3K‐dependent mechanism. In a final analogy utilizing another accepted vitamin D target, the immune system, Ferreira and colleagues^(^ ^161^ ^)^ have reported that 1,25(OH)_2_D/VDR advances tolerogenic human monocyte‐derived dendritic cells through stimulation of glucose metabolism via a combination of nongenomic 1,25(OH)_2_D/VDR activation of PI3K/Akt/mTOR signaling and genomic induction of *PI3KCG*, *PFKFB4*, and *C‐MYC*, with the gene products respectfully activating PI3K, glycolysis, and growth. Thus, through combined nongenomic and genomic signaling, 1,25(OH)_2_D/VDR dictates the creation and maintenance of the tolerogenic monocyte‐derived dendritic cell phenotype and its functions. This indicates that the immune system joins bone and brain in the group of vitamin D targets that appears to employ coupled nongenomic and genomic mechanisms of 1,25(OH)_2_D/VDR action.

## Disclosures

All authors have nothing to disclose.

## Author contributions

**Mark Haussler:** Conceptualization; data curation; formal analysis; funding acquisition; writing‐original draft; writing‐review and editing. **Sarah Livingston:** Data curation; investigation; methodology. **Zhela Sabir:** Data curation; investigation; methodology. **Carol Haussler:** Investigation; writing‐original draft; writing‐review and editing. **Peter Jurutka:** Conceptualization; data curation; formal analysis; funding acquisition; investigation; methodology; project administration; supervision; writing‐review and editing.

### Peer review

The peer review history for this article is available at https://publons.com/publon/10.1002/jbm4.10432.

## Supporting information


**Fig. S1.** Induction time‐course of mRNAs encoding bone‐expressed genes following treatment of rat osteocyte‐like UMR‐106 cells with 1,25(OH)_2_D. *(A) cyp24a1*, *(B) fgf23*, *(C) spp1*, *(D) nurr1*. Each value is the average of at least three experiments with triplicate biological replicates ± standard deviation.
