# Supplementary figures and images for: Vitamin D Receptor Mediates a Myriad of Biological Actions Dependent on Its 1,25‐Dihydroxyvitamin D Ligand: Distinct Regulatory Themes Revealed by Induction of Klotho and Fibroblast Growth Factor‐23
Source: JBMR Plus. 2020 Dec 3;5(1):e10432. doi: 10.1002/jbm4.10432 (PMC7839824; doi:10.1002/jbm4.10432)

## Slide 1
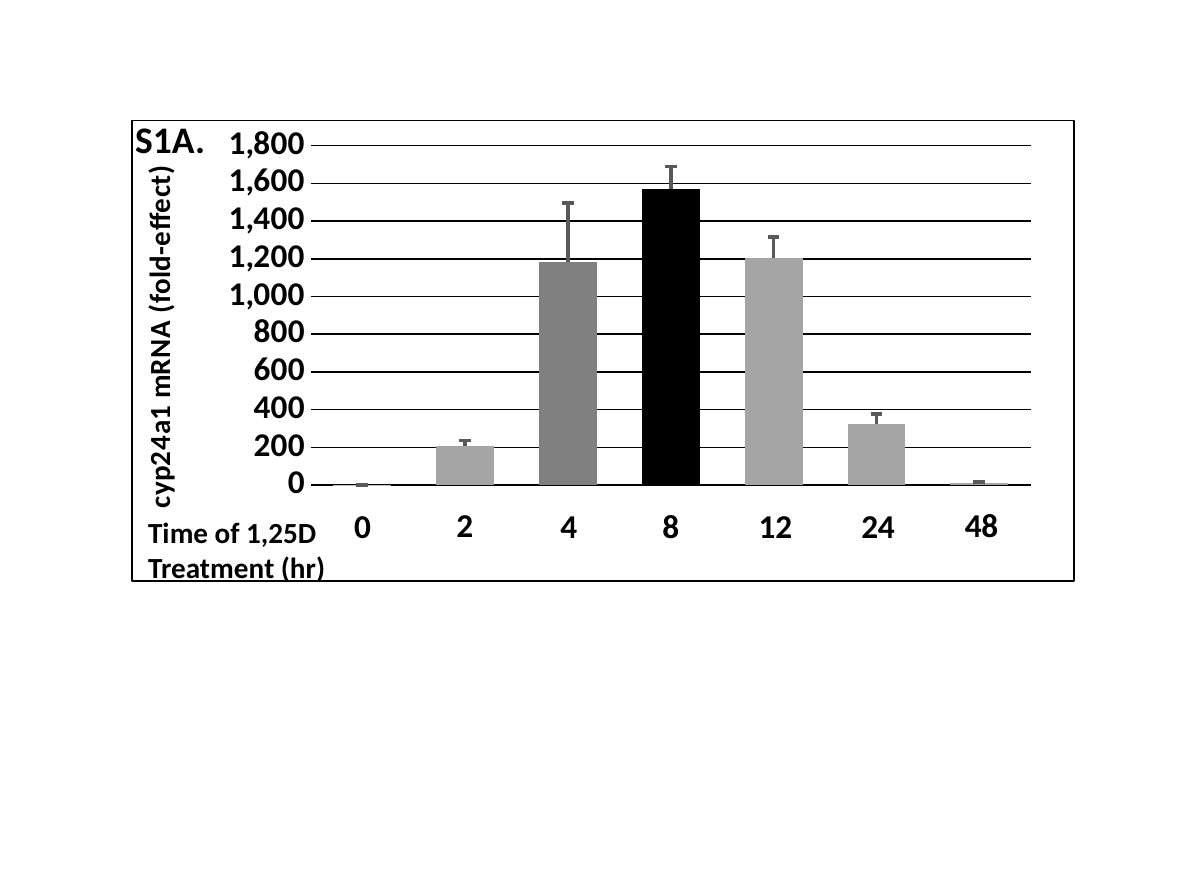

S1A.
### Chart
| Category | Ave |
|---|---|
| 0 hour | 1.0066666666666666 |
| 2 hour | 206.63333333333333 |
| 4 hour | 1181.1499999999999 |
| 8 hour | 1571.5199999999998 |
| 12 hour | 1203.0466666666666 |
| 24 hour | 325.0933333333333 |
| 48 hour | 14.273333333333333 |2
48
4
8
0
12
24
Time of 1,25D
Treatment (hr)

Supplement: Supplementary file 1 — Fig. S1. Induction time‐course of mRNAs encoding bone‐expressed genes following treatment of rat osteocyte‐like UMR‐106 cells with 1,25(OH)2D. (A) cyp24a1, (B) fgf23, (C) spp1, (D) nurr1. Each value is the average of at least three experiments with triplicate biological replicates ± standard deviation. [file JBM4-5-e10432-s001.zip › JBM4_10432_Figure S1A final 3 cyp24a1 mRNA.pptx]

## Slide 1
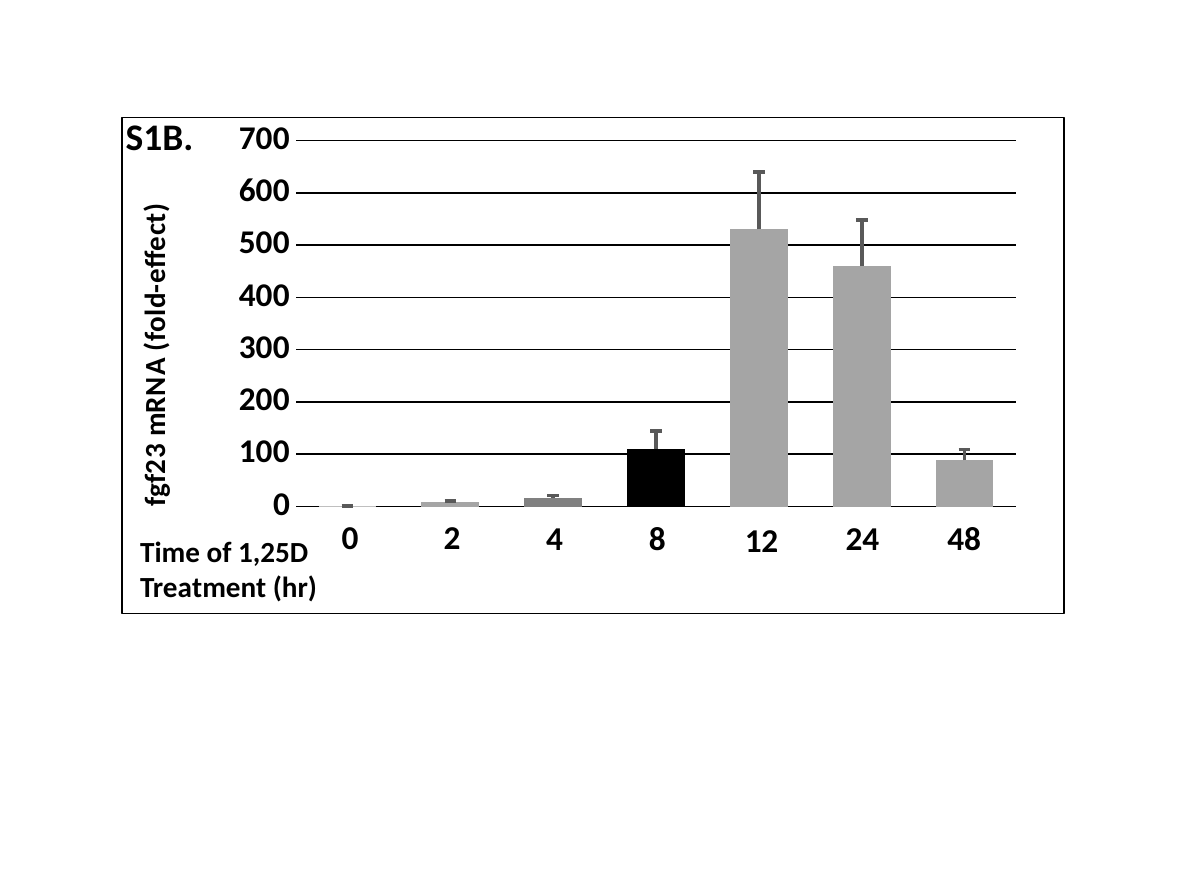

S1B.
### Chart
| Category | average |
|---|---|
| 0 hour | 1.03 |
| 2 hour | 8.763333333333334 |
| 4 hour | 17.19 |
| 8 hour | 109.50666666666666 |
| 12 hour | 530.2866666666667 |
| 24 hour | 460.0233333333333 |
| 48 hour | 88.92333333333333 |2
0
48
24
8
4
12
Time of 1,25D
Treatment (hr)

Supplement: Supplementary file 1 — Fig. S1. Induction time‐course of mRNAs encoding bone‐expressed genes following treatment of rat osteocyte‐like UMR‐106 cells with 1,25(OH)2D. (A) cyp24a1, (B) fgf23, (C) spp1, (D) nurr1. Each value is the average of at least three experiments with triplicate biological replicates ± standard deviation. [file JBM4-5-e10432-s001.zip › JBM4_10432_Figure S1B final 3 fgf23 mRNA.pptx]

## Slide 1
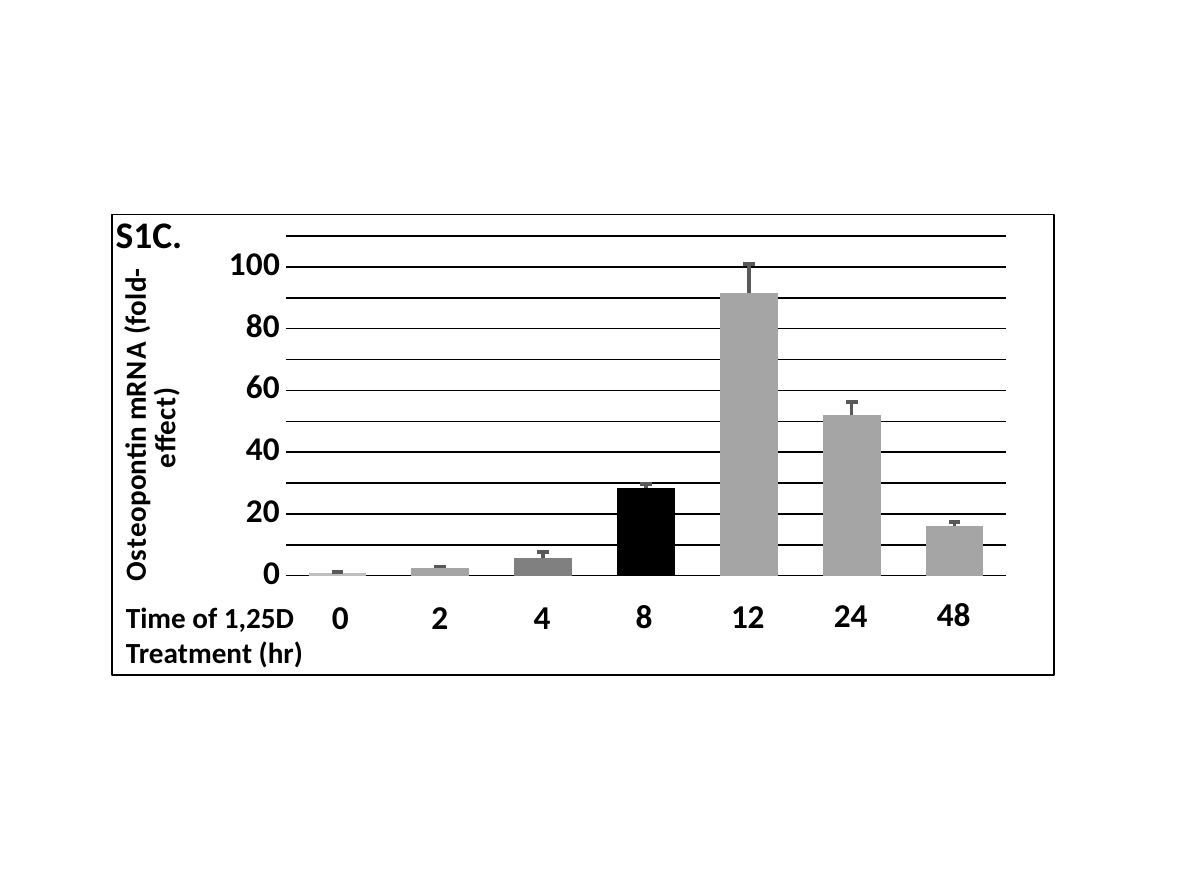

S1C.
### Chart
| Category | Average |
|---|---|
| 0 hour | 1.0033333333333332 |
| 2 hour | 2.5966666666666667 |
| 4 hour | 5.78 |
| 8 hour | 28.28 |
| 12 hour | 91.43333333333334 |
| 24 hour | 52.09666666666667 |
| 48 hour | 15.973333333333336 |48
24
12
8
0
2
4
Time of 1,25D
Treatment (hr)

Supplement: Supplementary file 1 — Fig. S1. Induction time‐course of mRNAs encoding bone‐expressed genes following treatment of rat osteocyte‐like UMR‐106 cells with 1,25(OH)2D. (A) cyp24a1, (B) fgf23, (C) spp1, (D) nurr1. Each value is the average of at least three experiments with triplicate biological replicates ± standard deviation. [file JBM4-5-e10432-s001.zip › JBM4_10432_Figure S1C Final osteopontin mRNA.pptx]

**S1D.**

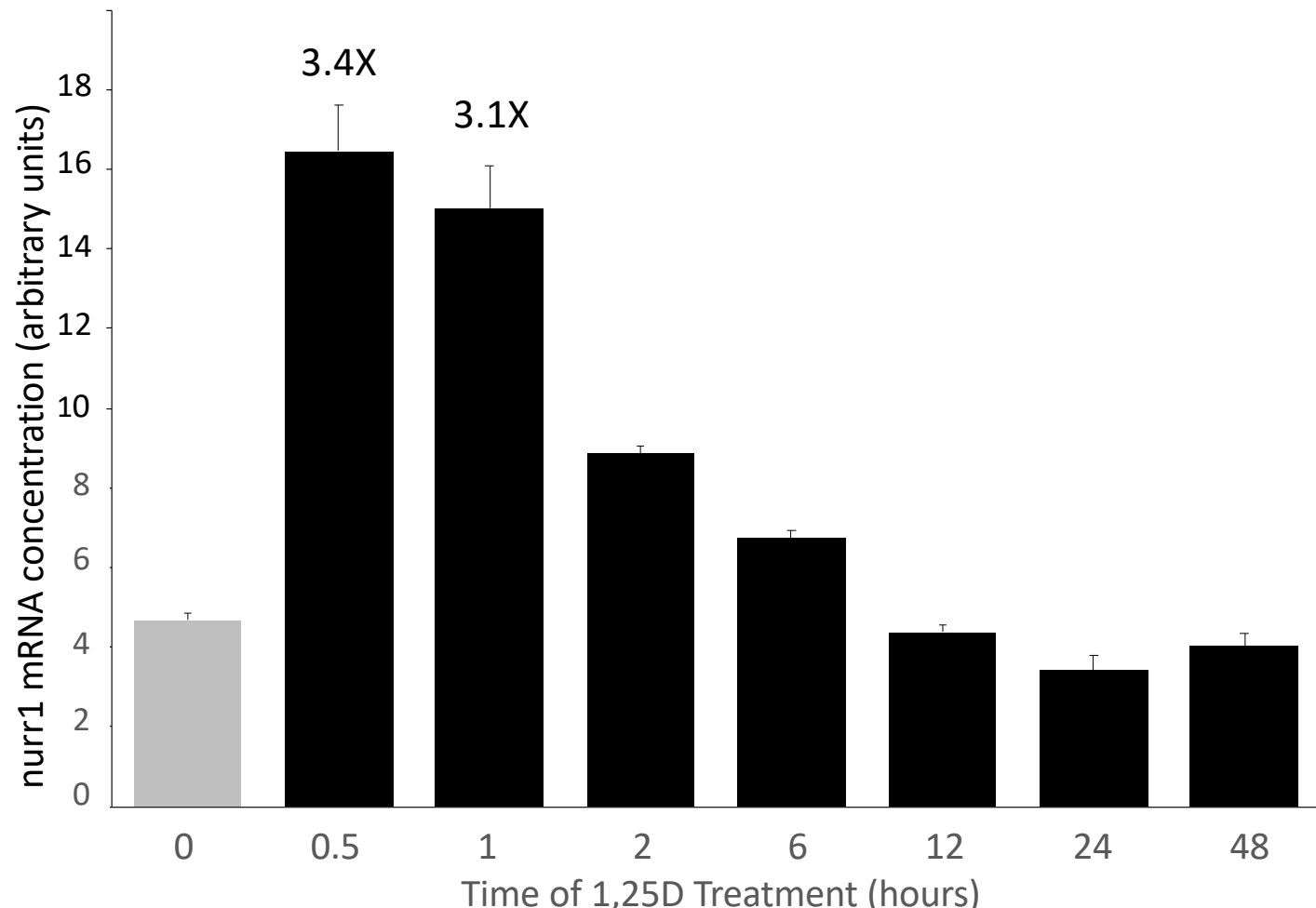

Supplement: Supplementary file 1 — Fig. S1. Induction time‐course of mRNAs encoding bone‐expressed genes following treatment of rat osteocyte‐like UMR‐106 cells with 1,25(OH)2D. (A) cyp24a1, (B) fgf23, (C) spp1, (D) nurr1. Each value is the average of at least three experiments with triplicate biological replicates ± standard deviation. [file JBM4-5-e10432-s001.zip › JBM4_10432_Figure S1D Final2 Time Course UMR-106 nurr1 1,25D timecourse.pdf]
